# Gene augmented nuclear-targeting sonodynamic therapy via Nrf2 pathway-based redox balance adjustment boosts peptide-based anti-PD-L1 therapy on colorectal cancer

**DOI:** 10.1186/s12951-021-01094-x

**Published:** 2021-10-29

**Authors:** Guoyun Wan, Xuheng Chen, Haijiao Wang, Shenglei Hou, Qian Wang, Yuanyuan Cheng, Qian Chen, Yingge Lv, Hongli Chen, Qiqing Zhang

**Affiliations:** 1 The Key Laboratory of Biomedical Material, School of Life Science and Technology, Xinxiang Medical University, 453003 Xinxiang, China; 2 School of Pharmacy, Tianjin Key Laboratory on Technologies Enabling Development of Clinical Therapeutics and Diagnostics (Theranostics), Tianjin Medical University, 300070 Tianjin, China; 3 Institute of Biomedical Engineering, The Second Clinical Medical College, Jinan University (Shenzhen People’s Hospital), 518020 Shenzhen, China; 4 Post-doctoral Scientific Research Station of Basic Medicine, Jinan Unviersity, 510632 Guangzhou, China

**Keywords:** Nuclear-targeting, Nrf2-siRNA, Sonodynamic therapy, Immune checkpoint blockade therapy, Colorectal cancer

## Abstract

**Background:**

Colorectal cancer is known to be resistant to immune checkpoint blockade (ICB) therapy. Sonodynamic therapy (SDT) has been reported to improve the efficacy of immunotherapy by inducing immunogenic cell death (ICD) of cancer. However, the SDT efficacy is extremely limited by Nrf2-based natural redox balance regulation pathway in cancer cells in response to the increased contents of reactive oxygen species (ROS). Nuclear-targeting strategy has shown unique advantages in tumor therapy by directly destroying the DNA. Thus it can be seen that Nrf2-siRNA augmented nuclear-targeting SDT could boost ICB therapy against colorectal cancer.

**Results:**

The nuclear-targeting delivery system TIR@siRNA (TIR was the abbreviation of assembled TAT-IR780) with great gene carrier capacity and smaller diameter (< 60 nm) was designed to achieve the gene augmented nuclear-targeting SDT facilitating the anti-PD-L1 (programmed cell death-ligand-1) therapy against colorectal cancer. In CT26 cells, TIR@siRNA successfully delivered IR780 (the fluorescent dye used as sonosensitizer) into cell nucleus and Nrf2-siRNA into cytoplasm. Under US (utrasound) irradiation, TIR@siRNA notably increased the cytotoxicity and apoptosis-inducing activity of SDT through down-regulating the Nrf2, directly damaging the DNA, activating mitochondrial apoptotic pathway while remarkably inducing ICD of CT26 cells. In CT26 tumor-bearing mice, TIR@siRNA mediated gene enhanced nuclear-targeting SDT greatly inhibited tumor growth, noticeably increased the T cell infiltration and boosted ^D^PPA-1 peptide-based anti-PD-L1 therapy to ablate the primary CT26 tumors and suppress the intestinal metastases.

**Conclusions:**

All results demonstrate that TIR@siRNA under US irradiation can efficiently inhibit the tumor progression toward colorectal CT26 cancer in vitro and in vivo by its mediated gene augmented nuclear-targeting sonodynamic therapy. Through fully relieving the immunosuppressive microenvironment of colorectal cancer by this treatment, this nanoplatform provides a new synergistic strategy for enhancing the anti-PD-L1 therapy to ablate colorectal cancer and inhibit its metastasis.

**Graphical Abstract:**

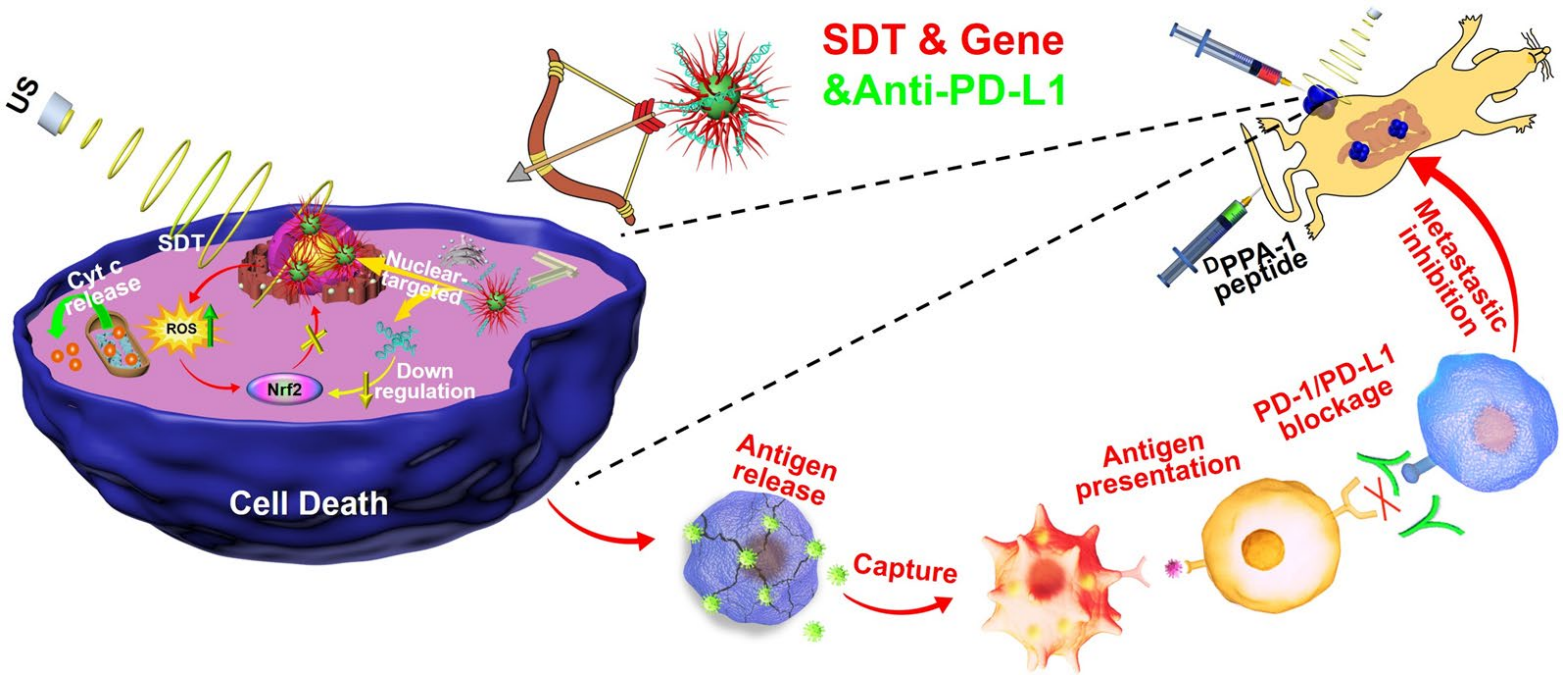

**Supplementary Information:**

The online version contains supplementary material available at 10.1186/s12951-021-01094-x.

## Introduction

Colorectal cancer (CRC) has become the third most common and fourth most lethal cancer type worldwide, accounting for 10% of cancer-related deaths annually [1]. The 5-year survival rate for CRC patients in the early stage is approximately 90%, whereas it is as low as 13.1% in patients in the advanced stage [2]. Currently, surgical resection remains the treatment of choice for early CRC in the clinic, although it is extremely difficult for patients with low rectal cancer to maintain the integrity of anal function. In addition, for advanced CRC patients with distant metastasis, chemotherapy is regarded as the main treatment option, but it comes with serious side effects [3, 4]. Fortunately, immunotherapy based on immune checkpoint blockade (ICB) to interfere with programmed cell death-1/programmed cell death-ligand 1 (PD-1/PD-L1) has attracted tremendous interests in the field of cancer treatment and has shown great therapeutic effects in recent years. PD-1 is a famous immune checkpoint receptor that is expressed on activated T cells, while solid tumor cells overexpress PD-L1, which in turn binds to PD-1 to restrain T cell proliferation and activation [5, 6]. This mechanism has driven the development of receptor blockers against PD-1/PD-L1, such as monoclonal antibodies and therapeutic peptides (AUNP-12 [7, 8], CLP002 [9], ^D^PPA-1 [10, 11]). Nivolumab and pembrolizumab, which work against PD-1, have been approved by the Food and Drug Administration (FDA) for the treatment of metastatic CRC [12, 13]. However, approximately 95% of CRC patients do not respond to PD-1/PD-L1 blockade therapy due to the tumor immunosuppressive microenvironment; thus, only a subset of CRC patients can benefit from it [14, 15]. Therefore, in-depth investigations into strategies to increase the response rate of ICB therapy, especially combinatory therapeutic approaches, have been called for.

Accumulating studies have indicated that various treatment methods, such as chemotherapy [16, 17], photodynamic therapy (PDT), photothermal therapy (PTT) [18–20] and chemodynamic therapy (CDT) [21, 22], can both kill tumors and activate the immune system by releasing tumor-associated antigens (TAAs) to stimulate the host’s specific immune response to cancer, thus assisting ICB therapy [23, 24]. For example, PDT utilizes photosensitizers and light to convert oxygen into reactive oxygen species (ROS) [25, 26], which can induce immunogenic cell death (ICD) in cancer cells by exposing and releasing damage-associated molecular patterns (DAMPs) and subsequently convert the immunosuppressive microenvironment (“cold” tumors) into an immune-activated microenvironment (“hot” tumors) [27–30]. This produced ICD effect offers a new dimension to facilitate ICB therapy by increasing the infiltration of DCs and T cells. Sonodynamic therapy (SDT), a new cancer treatment modality, has the same mechanism of action as PDT against tumors but has overcome the PDT limitation of penetration depth [31–33]. Therefore, SDT is becoming a promising treatment for curing cancers and is especially suitable for large solid or deep-seated tumors due to its effective irradiation of sonosensitizers to produce ROS. In recent decades, various sonosensitizers have been developed for SDT, such as inorganic TiO_2_ nanoparticles, crystalline silicon, polyhydroxy fullerenes, organic porphyrins, and NIR fluorescence dyes (ICG, IR780, IR825, etc.) [24, 31–36]. Moreover, inspired by the ICD effects of PDT and the fact that ultrasound can penetrate deeper than light, we hypothesized that deeply penetrating irradiated sonosensitizers would greatly assist ICB therapy while killing deep-seated tumors.

However, redox regulation pathways that naturally exist in tumor cells can attenuate oxidative pressure to partly impede the curative effects of PDT or SDT. For instance, the nuclear factor (erythroid-derived 2)-like 2 (Nrf2)-mediated classical deoxidation signaling pathway plays an important role in maintaining normal redox levels and has been found to result in tumor cell resistance to PDT or SDT by continually consuming ROS. Therefore, Nrf2-siRNA can be applied as a helper for SDT to enhance its antitumor effects by downregulating Nrf2 protein expression and inhibiting ROS detoxification [37]. Therefore, the codelivery of Nrf2-siRNA into tumor cells with a sonosensitizer and efficient transfection will greatly enhance SDT efficacy. In previous studies, researchers developed gene delivery systems based on TAT peptide-modified liposomes [38] and micelles [39] and achieved the efficient and selective transfection of the desired genes in tumor cells based on their strong positive charge. The positively charged TAT peptide can complex with anionic genes and enhance internalization into tumor cells through their excellent cell-penetrating efficiency. In addition, a previous study revealed that the TAT peptide could alter the permeability of lysosomes, which could promote gene escape from lysosomes due to its high positive charge [40], and thus accomplishing gene transfection.

Therapeutic efficacy against tumors is usually determined by the delivery efficiency of the drugs to their final targets to a large extent. Organelle targeting also plays the important role in the targeted treatment to amplify its therapeutic efficacy. Recently, various organelle-targeted nanosystems have developed to transport drugs into specific organelles (the nucleus [41], mitochondria [35], lysosomes [42], endoplasmic reticulum [43], and Golgi apparatus [44]) in tumor cells have attracted great attention. The specially designed nanomaterials can either effectively deliver drugs to the proper target or directly destroy the subcellular structure, thus further induce the cell death, recurrence prevention, side effects reduction and drug resistance remission depending on different signal based pathways [45]. Among them, the cell nucleus, which stores most genetic materials, have been reported to be the main site of action for most therapeutic agents like chemotherapeutic drugs, free radicals, heat or genes. For instance, nucleus-targeting PTT can closely and directly “burn” genetic materials, thus inhibiting the proliferation of cancer cells. It is therefore highly expected that cell nucleus-targeting nanocarriers will provide a more effective strategy than other random-or subcellular-distributed nanocarriers to benefit cancer treatment with precise nanomedicine. The TAT peptide, a classical cell-penetrating peptide, has excitingly been reported to possess strong nucleus-targeting properties and achieve the nucleus-targeted delivery of photosensitizers. Previously, an Ir-HSA nanoparticle, as a photosensitizer, successfully achieved nuclear targeting for PDT against cancer [46]. However, there is still a lack of research on the development of nuclear-targeting sonosensitizers for nuclear-targeting SDT in cancer treatment.

**Scheme 1:**
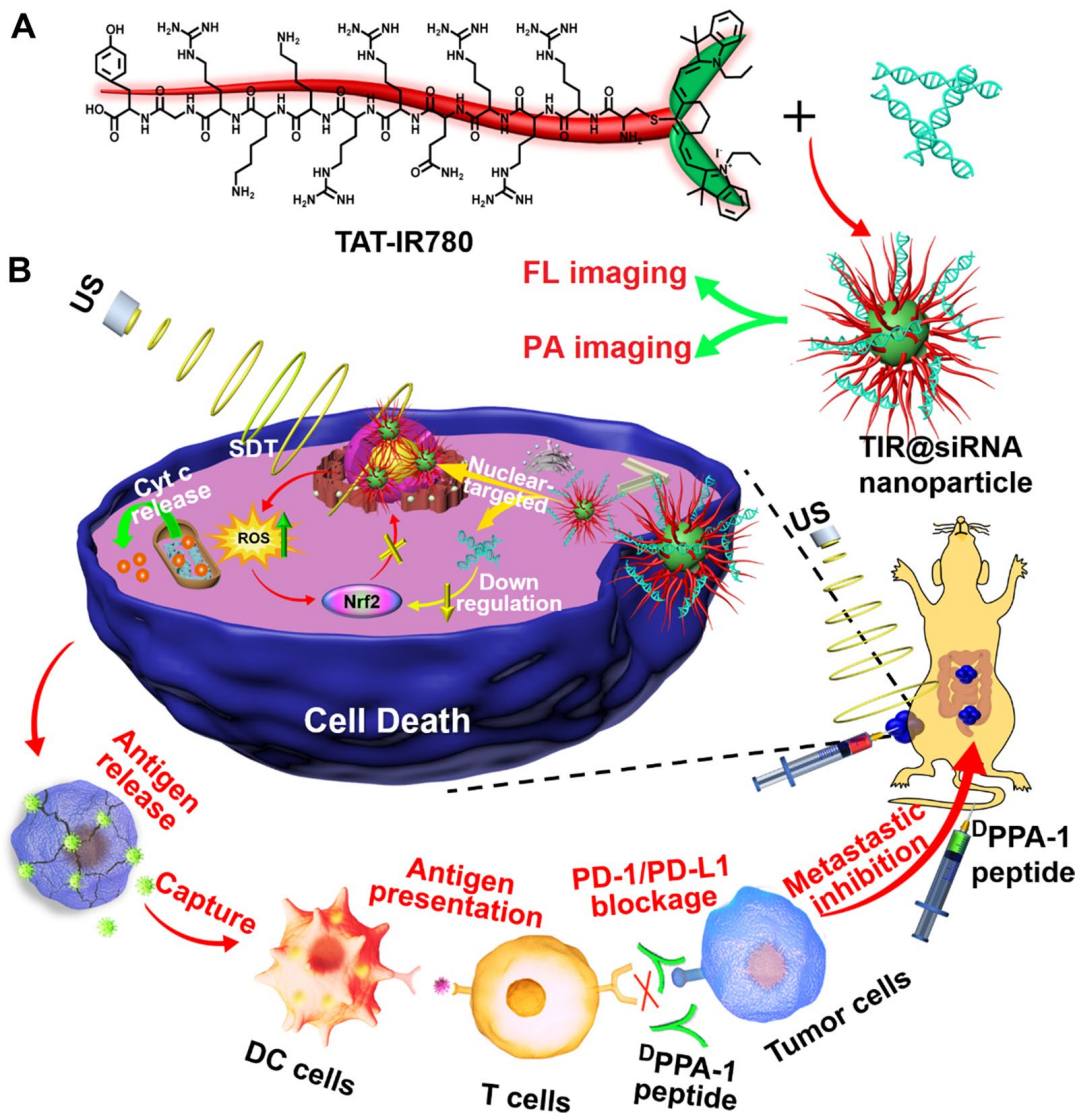
Illustrations for **A** preparation of TIR@siRNA nanoparticles and **B** their functional mechanisms against colorectal cancer via gene enhanced nuclear-targeted SDT boost anti-PD-L1 therapy in vitro and in vivo

To this end, we designed a nuclear-targeting sonosensitizer based on the TAT peptide coupled IR780 (TAT-IR780) to load Nrf2-siRNA with the hopes of achieving gene-augmented nuclear-targeting SDT against colorectal cancer and further boosting peptide-based anti-PD-L1 therapy against colorectal metastasis. As outlined in Scheme 1 A, TAT-IR780 and Nrf2-siRNA self-assembled through electrostatic interactions to form nanoparticles denoted as TIR@siRNA. Scheme 1B displays the in vivo function and mechanism of TIR@siRNA against CRC. After intratumoral injection, the TIR@ siRNA nanoparticles could penetrate and adhere to the tumor tissues for a longer time due to the strong positive charge of the TAT peptide. Additionally, TIR@siRNA shows great fluorescence and photoacoustic imaging efficiencies due to the optical properties of IR780. After cellular internalization, TIR@siRNA enhanced the cellular uptake of IR780 and Nrf2-siRNA, escaped from the lysosomes and translocated into the nucleus. Upon US irradiation, TIR exerted its sonodynamic activity to trigger the generation of ROS, which can directly kill cancer cells. Moreover, Nrf2-siRNA suppressed the upregulation of Nrf2 after SDT, blocked the redox balance regulation pathway and enhanced the antitumor effects of SDT mediated by TIR by maintaining a high level of intracellular ROS. Furthermore, this gene-enhanced nuclear-targeting SDT strategy can induce ICD to improve the immunosuppressive microenvironment, thus boosting ^D^PPA-1 peptide-based anti-PD-L1 therapy of CRC and inhibiting its intestinal metastasis through activation of the systemic immune response.

## Methods

### Materials

Cysteine terminated TAT peptide (YGRKKRRQRRRC-NH2) and ^D^PPA-1 peptide were synthesized by ChinaPeptides (Shanghai, China). IR780 iodide was purchased from Sigma-Aldrich (St. Louis, USA). Nrf2-siRNA and FITC-labeled Nrf2-siRNA (FITC-siRNA) were synthesized by GenePharma Biotech (Suzhou, China). DCFH-DA, SOSG, Annexin-V-FITC/7AAD apoptosis kit and LIVE/DEAD cell imaging kit were obtained from Meilun Biotechnology (Dalian, China). Lysotracker red and Mitotracker green (M7514) fluorescent probes were bought from Thermo Fisher Scientific (Hudson, NH, USA). Methylthiazolyldiphenyl-tetrazolium bromide (MTT) and 9,10-Anthracenediyl-bis(methylene) dimalonic acid (ABDA) were purchased from Heowns Biochem Technology (Tianjin, China). Monoclonal antibodies against γ-H2AX, Nrf2 and cytochrome c were provided by Abcam (Cambridge, MA, USA). Monoclonal antibodies against HSP70, Ki67, CD11c, CD4, CD8 and FITC-labeled monoclonal antibody against calreticulin were obtained from Bioss Biotechnology (Beijing, China)., FITC-labeled and PE-labeled secondary antibodies were supplied by Shandong Sparkjade Biotechnology Co., Ltd. (Shandong, China). The PE-labeled CD44 and APC-labeled CD62L monoclonal antibodies were purchased from eBioscience (San Diego, USA).

Mouse colorectal cancer cell line CT26 was purchased from National Biomedical Laboratory Cell Resource Bank (Beijing, China). The cells were cultured in RPMI 1640 medium containing 10% FBS and 1% penicillin-streptomycin and placed in a 5% CO_2_ incubator at 37 °C.

### Synthesis and characterization of TAT-IR780

TAT-IR780 was synthesized by a substitution reaction of sulfhydryl group in TAT peptide and chlorine atom in IR780 according to our previous report [47]. Briefly, 120 mg TAT peptide, 40 mg IR780 iodide and 15 µL TEA were dissolved in DMSO and reacted at 40 °C with N_2_ protection for 72 h. At the end of the reaction, the reaction completion was detected by thin-layer chromatography (TLC) with the eluting solvent of methanol/chloroform (3:1, v/v) while the TAT peptide mixed IR780 was conducted as the control. Then the mixture was dialyzed in deionized water for 24 h in the dark with a dialysis bag (1000 Da) to remove the unreacted IR780 and TAT peptide. Finally, the dark green powder of TAT-IR780 was obtained by the lyophilization of dialysate. The molecular weight of TAT-IR780 was characterized by an API 150EX mass spectrometer (Agilent, USA).

### Preparation and characterization of TIR@siRNA nanoparticles

Typical agarose gel electrophoresis was applied to investigate the interaction between TAT-IR780 and Nrf2-siRNA through the charge interaction. In detail, TAT-IR780 (dissolved in DMSO) were transitorily vortexed with 1000 ng siRNA at different ratios of N/P (0/1, 8/1, 12/1, 16/1, 32/1, 64/1) in DEPC water and then stand at room temperature for 30 min. After loading buffer added, the samples were loaded onto an agarose gel (3%) and then electrophoresed in TAE buffer at 130 V for 15 min by a JUNYISPAT electrophoresis device (Junyi-Dongfang, Beijing, China). Finally, the siRNA bands were imaged via a Gel Doc-It 310 imaging system (UVP, CA, USA). The zeta potentials of TAT-IR780 only and the nanocomplex with the N/P ratios of 32/1, 64/1 were measured by a Malvern laser particle analyzer (Worcestershire, UK).

N/P ratio of 64/1 was chosen to prepare TIR@siRNA nanoparticles while the mass ratio of IR780 to siRNA was about 4:1. Firstly, TAT-IR780 was dissolved in DMSO then diluted with DEPC water to be 800 µg/mL (on the basis of IR780) and Nrf2-siRNA was dissolved in DEPC water at the concentrations of 200 µg/mL, respectively. Then the equal volume of TAT-IR780 solution was quickly added into the Nrf2-siRNA under gentle vortex for 10 s. Followed by standing in dark environment and room temperature for 30 min, the nanoparticles called TIR@siRNA were successfully obtained. The TIR nanoparticles were prepared with the same method as there is no siRNA added.

The morphologies of TIR and TIR@siRNA were observed by a HT7700 transmission electron microscope (TEM, Tokyo, Japan) and their particle size, size distribution were detected by a Malvern laser particle analyzer. Besides, the optical stability of free IR780, TIR and TIR@siRNA during 5 d storage in water preventing from light were also studied. The UV-Vis spectrums of the samples at IR780 concentration of 10 µg/mL were recorded everyday by an UV-2450 spectrophotometer (Shimadzu, Japan). Meanwhile, the fluorescence intensity of them was also measured by a RF-5301 fluorescence spectrometer (Shimadzu, Japan) with the Ex = 780 nm and Em = 810 nm at IR780 concentration of 2 µg/mL. Besides, the fluorescence changes were visually imaged by an IVIS in vivo imager (PerkinElmer, Waltham, USA) on 1st and 5th day.

### Evaluation of the sonobleaching

The solutions of free IR780, TIR or TIR@siRNA containing 10 µg/mL IR780 were irradiated with US (1 MHz, 1.0 W/cm^2^, 50 % duty cycle) for different time intervals and the UV absorbance attenuation were monitored by UV-Vis spectrophotometer to evaluate the sound bleaching effect of US to IR780.

### In vitro ROS generation

The commercial probe ABDA was used as an indicator to evaluate the generation of ROS induced by TIR@siRNA under US irradiation according to our previous report [48]. Briefly, 100 µM ABDA was added to 25 µg/mL TIR@siRNA dispersion (on the basis of IR780) and then the mixture was irradiated with US (1 MHz, 1.0 W/cm^2^, 50% duty cycle) for 10 min. During the irradiation, the UV-Vis spectrums of ABDA were recorded by UV-Vis spectrophotometer at different time intervals.

### Cellular uptake and subcellular localization measurements

10^5^ CT26 cells·well^−1^ seeded on 12-well chambered cover glass were incubated with FITC-Nrf2-siRNA, free IR780, TIR and TIR@FITC-Nrf2-siRNA for 4 h. The concentrations of IR780 and siRNA were 6 µg/mL and 1.5 µg/mL, severally. After fixed with paraformaldehyde and stained with DAPI for nuclei staining, the cells were imaged by a TCS SP8 confocal microscopy (Leica, Germany). Besides, flowcytometry was applied for the quantitative analysis of the uptake of TIR@FITC-Nrf2-siRNA by CT26 cells. Briefly, the cells seeded on a 12-well plate were treated with FITC-Nrf2-siRNA, free IR780, TIR and TIR@FITC-Nrf2-siRNA at IR780 and siRNA concentrations of 2 µg/mL and 0.5 µg/mL for 4 h and then collected to be analyzed via a Guava easyCyte 5 flow cytometer (Merck Millipore, Darmstadt, Germany).

### Penetration of TIR@siRNA nanoparticles into colorectal tumor-like spheroids

The tumor-like spheroids were firstly constructed according to a classical method refer to a previous research [49]. In brief, 3000 CT26 cells in 200 µL complete 1640 medium were dropped vertically into an agarose-coated 96-well plate and further cultured for 5 days. The medium was changed once on the 3rd day. Next, the spheroids with the same size were selected to incubate with free IR780, TIR and TIR@siRNA at IR780 concentration of 15 µg/mL for 4 and 12 h. After rinsed with PBS twice, the fluorescence intensity of CT26 spheroids was imaged by confocal microscopy. In addition, the CT26 spheroids after 12-h incubation of IR780, TIR and TIR@siRNA were intuitively observed with a digital camera.

### Acridine orange lysosomal permeabilization experiments

10^5^ CT26 cells/well were seeded onto 15-mm confocal dishes and cultured for 24 h, then the cells were treated with IR780, TIR and TIR@siRNA at IR780 concentration of 6 µg/mL for 4 h. After that, the cells were rinsed with PBS for twice and stained with acridine orange for 20 min at 37 ℃. Finally, after washed with PBS, the cells were imaged via confocal microscopy and analyzed by flow cytometry to assess the fluorescence change of acridine orange.

### Lysosome escape experiments

10^5^ CT26 cells/well were seeded onto 15-mm confocal dishes and cultured for 24 h. After incubated with TIR@FITC-Nrf2-siRNA at the siRNA concentration of 1.5 µg/mL for 2 and 4 h, the cells were processed stained with LysoTracker Red and Hoechst 33342 for 20 min one by one. Finally, the cells were washed with PBS and then imaged by confocal microscopy to observe the fluorescence of lysosomes and FITC-Nrf2-siRNA. Besides, the software Image J was used to analyze the colocalization of siRNA and lysosomes.

### Cytotoxicity assessment

MTT assay was used to assess the cytotoxicity of TIR@siRNA under US irradiation. Briefly, CT26 cells were randomly seeded into 96-well plates at a density of 5000 cells per well and cultured for 24 h. Different concentrations of free IR780, TIR and TIR@siRNA were added to the wells. After 4-h incubation, some cells were exposed to US irradiation at the power intensity of 1.0 W/cm^2^ for 5 min (1 MHz, 50 % duty cycle), all cells were cultured for another 20 h. Afterward, 10 µL MTT solution (5 mg/mL) was added to each well and the produced formazan crystals were dissolved by DMSO after 3 h. Finally, the absorbance values of each well were determined by a microplate reader (Molecular Devices, California, USA) at 490 nm.

To visually evaluate the cytotoxicity of TIR@siRNA mediated SDT, the LIVE/ DEAD kit was further used to stain the above cells treated with free IR780, TIR and TIR@siRNA with/without US irradiation based on IR780 concentration of 4 µg/mL. Through this assay, the live cells were stained with calcein-AM while the dead cells were stained with PI for 30 min, and thus could be observed through a fluorescent microscope (Olympus, Tokyo, Japan).

### Cell apoptosis assay

CT26 cells were randomly implanted in 12-well plates and allowed to adhere for 24 h at a density of 1.5 × 10^5^ cells per well. Then the cells were severally incubated with free IR780, TIR and TIR@siRNA at IR780 concentration of 4 µg/mL for 4 h, and then some cells were received the US irradiation (1 MHz, 1.0 W/cm^2^, 50 % duty cycle) for 5 min. After further 20-hour incubation, the cells were stained with annexin V-FITC and 7AAD for 5 min in the dark. Finally, the cells were collected and analyzed by a flow cytometry.

### In vitro DNA damage study

H2AX phosphorylation was analyzed as a marker of DNA damage of TIR@siRNA mediated nuclear-targeting SDT. Briefly, the CT26 cells seeded on 12-well glasses tips were received the same treatments as described above. Then the cells were fixed and permeated before treated with blocking reagent. Afterward, the cells were incubated with primary antibody anti-γH2AX (1:250), followed by secondary antibody FITC-conjugated goat anti-rabbit antibody (1:400), and DAPI for nucleus staining. Finally, the cells were photographed by a confocal microscope.

### Intracellular ROS generation

Intracellular ROS generation by gene augmented SDT treatment was assessed by the ROS detection kit (DCFH-DA as a probe). CT26 cells seeded in 12-well plate were incubated for 4 h with free IR780, TIR and TIR@siRNA at IR780 concentration of 4 µg/mL, then the cells were exposed to US irradiation at the power dose of 1.0 W/cm^2^ for 5 min (50% duty cycle). After further cultured for 12 h, the cells were treated with DCFH-DA for 30 min and subsequently observed with fluorescence microscopy (Ex: 488 nm) followed DAPI staining, and as well as quantified by flow cytometry. The DCFH-DA concentrations used for fluorescence observation and flow cytometry analysis were 20 µM and 5 µM in PBS, respectively.

### Expression analysis of cellular Nrf2 by immunofluorescence and western blotting

Immunofluorescence technique was firstly contributed to visually assess the Nrf2 expression in CT26 cells after various treatments. Briefly, the CT26 cells seeded on 12-well glass tips were treated separately with free IR780, TIR and TIR@siRNA for 4 h on the basis of IR780 concentration of 4 µg/mL and Nrf2-siRNA concentration of 2 µg/mL. Then some of the cells were treated with US irradiation (1 MHz, 1.0 W/cm^2^, 50% duty cycle) for 5 min. After further cultured for 20 h, the cells were processed with fixation, punction, block and incubation with polyclonal anti-Nrf2 antibody overnight. Followed by staining with FITC-conjugated secondary antibody and DAPI, the cells were photographed via a confocal microscope.

The western blotting assay was further used to evaluate the expression of Nrf2 in response to various treatments. The CT26 cells cultured in 6-well plates received the same treatments as described above. Then the cells were collected and lysed with RIPA buffer. Next, equal amounts (30 µg) of total protein were subjected to SDS-PAGE and transferred onto PVDF membranes, and then blocked with 5% milk for 1 h. The membranes were probed with antibodies against Nrf2 and glyceraldehyde-3-phosphate dehydrogenase (GAPDH) to ensure equal protein loading according to the manufactures’ instructions. Finally, the membranes were treated with HRP-linked secondary antibody and the immune-reactive bands were visualized using a G-Box chemiluminescence image capture system (Syngene, Cambridge, UK).

### Mitochondrial membrane potential detection

CT26 cells were cultured in 12-well plates overnight and then treated with PBS, IR780, TIR, TIR@siRNA at an equivalent dose of 4 µg/mL. 4 h later some cells were irradiated with US (1 MHz, 1.0 W/cm^2^, 50% duty cycle) for 5 min. Followed by further incubation of 24 h, the cells were stained with JC-1 as a fluorescence probe at a concentration of 10 µM for 30 min. For CLSM, cells were stained with DAPI and observed directly. For flow cytometry, cells were trypsinized and collected for analysis.

### Observation of cytochrome c (Cyt c) release

CT26 cells seeded on 12-well plates with glass tips overnight and received the same treatments as above JC-1 staining experiment. Then the cells were processed with Mitotracker Green (1 µM), cold methanol, 0.2% Triton X-100 and 3% BSA. After being successively stained with primary anti-Cyt c antibody (1:250), PE-conjugated secondary antibody (1:400) and DAPI, the slides were observed via confocal microscopy. The decolocalization of red and green fluorescence indicates the release of Cyt c from mitochondria.

### Detection of ICD effects induced by gene augmented SDT

To determine SDT-induced ICD of CT26 cells, surface exposure of calreticulin (CRT) and intracellular expression of heat shock protein 70 (HSP70) were assessed via immunofluorescence technique. In detail, the CT26 cells seeded on 25-mm glass tips were treated with PBS, US irradiation, TIR@siRNA, TIR@siRNA combined US irradiation (1 MHz, 1.0 W/cm^2^, 50% duty cycle) at IR780 concentration of 4 µg/mL after 4-h co-incubation, and followed by further culturing for 12 h. For CRT exposure detection, the treated cells were directly stained with FITC-labeled primary anti-calreticulin antibody after blocked with 3% BSA and then fixed with cold methanol. For HSP70 expression measurement, the cells were processed with 4% PFA, 0.2% Triton X-100, 3% BSA, primary anti-HSP70 antibody and PE-conjugated secondary antibody. After stained with DAPI, all cells were photographed via a confocal microscope.

### In vitro BMDCs recruitment

For the BMDCs recruitment, the lysates used as tumor antigens were firstly extracted from CT26 cells with various treatments. Briefly, CT26 cells were seeded into 12-well plates at a density of 10^5^ cells per well and further cultured for 24 h. Then the cells were received the same treatments as above evaluation of ICD effects and further cultured for 20 h. The culture media were collected and removed the debris by centrifugation at 2000 rpm for 5 min, and the supernatants were used as the lysates.

Next, the BMDCs were harvested from the bone marrow of BALB/c mice as previously reported [50]. In detail, BM progenitors isolated from bone marrow were cultured in RPMI 1640 total medium with murine granulocyte-macrophage colorectaly-stimulating factor (GM-CSF) (20 ng/mL) (PeproTech, USA) and interleukin-4 (IL-4) (10 ng/mL) (PeproTech, USA) at 37 °C in a humidified incubator with 5% CO_2_ for 5–7 days to generate immature BMDCs.

Finally, the transwell migration system was conducted to investigate the recruitment of BMDCs induced by the released tumor antigens of CT26 cells induced by SDT using 12-well Transwell chambers with 3.0 μm pore size filters. The lysates derived from CT26 cells after various treatments were added into the lower chambers and 5 × 10^5^ BMDCs were planted into the upper chamber. After further co-culture for 24 h, BMDCs in the lower layer were collected and stained with 5 µM calcein-AM. Then the migrated BMDCs were observed via a fluorescence microscope (Olympus, Tokyo, Japan) and four perspectives were randomly chosen for counting analysis.

### Establishment of subcutaneous and orthotopic colorectal tumor models

Female BALB/c mice (5-6 weeks) were bought from SPF Biotechnology (Beijing, China) and maintained in a bacteria-free environment at 22 °C with Sterile water administration. Subcutaneous CT26 tumor-bearing mice were constructed by injected CT26 cells into the right hips of the mice at 1 × 10^6^ per mice. Orthotopic colorectal metastatic tumor models were established according to a published research [51]. Briefly, the mice were firstly anesthetized by intraperitoneal injection of chloral hydrate, then a 1 cm-surgical wound on the abdomen was cut to make the cecum expose on the sterile gauze through surgical forceps. 5 × 10^5^ CT26 cells dispersed in 25 µL mixture of PBS and Matrigel matrix at a ratio of 1:1 was injected into the submucosal layer of the cecum by using the 29 G BD Insulin Syringe and then the needle inlets were locked with 7-0 silk sutures to prevent leakage. Finally, the cecum was gently returned to the original location and the cecotomy was closed with 7-0 silk sutures. All animal experiments were performed under the guiding of the Institutional Animal Care and Use Committee at Xinxiang Medical University.

### In vivo retention and biodistribution assessment

In vivo retention and biodistribution of TIR@siRNA in subcutaneous CT26 tumor-bearing mice were assessed using in vivo imaging system. The mice were intratumorally injected with IR780, TIR and TIR@siRNA, respectively. Next, all mice were imaged with an IVIS imaging system at predetermined times and 20 µL whole blood collected from tail vein was diluted to 20 µL EDTA anticoagulant and followed imaged at the same time points. After 48 h post administration, the tumors and major organs were harvested from mice for further fluorescence imaging.

### In vitro and in vivo photoacoustic (PA) imaging of TIR@siRNA

For in vitro PA imaging, different concentrations (0 µg/mL, 10 µg/mL, 30 µg/mL, 50 µg/mL on the basis of IR780) of IR780 and TIR@siRNA solutions were added into photoacoustic catheter followed by exposing to a photoacoustic probe, then the PA signals under 808 nm were collected by Endra Nexus 128 PA scanner (Ann tbor, MI). As for the in vivo PA imaging, the tumor-bearing mice were intratumorally injected with 20 µg IR780 or TIR@siRNA. The PA signals in tumor tissues were captured at the predetermined time points post administration.

### In vivo assessment of SDT efficacy and mechanisms against CT26 cancer

To evaluate SDT efficacy of TIR@siRNA in vivo, a fluorescence probe SOSG was used to measure the intratumoral ROS generation. In detail, CT26 tumor-bearing mice were intratumorally injected with normal saline, free IR780, TIR and TIR@siRNA containing SOSG (50 µM) at IR780 and Nrf2 siRNA dosages of 2 mg/kg and 0.5 mg/kg, respectively. 4 h later, some mice were exposed to US irradiation (1 MHz, 2.0 W/cm^2^, 50% duty cycle) for 5 min and the tumor tissues were collected for cyro-section at 12 h post administration. Subsequently, the obtained sections were photographed with a fluorescence microscope after stained with DAPI. Furthermore, the tumor tissues were also made as paraffin-sections and processed with immunohisto-chemistry staining of Nrf2 and γ-H2AX to evaluate the SDT mechanisms against CT26 cancer.

### In vivo assessment of antitumor efficacy of gene augmented SDT

At one week post the implantation of the subcutaneous CT26 tumor model, the mice were randomly divided into six groups (n = 5 per group) and then intratumorally injected with 100 µL of PBS, free IR780, TIR and TIR@siRNA at IR780 dosage of 2.0 mg/kg and Nrf2-siRNA dosage of 0.5 mg/kg on day 0 and day 4. At 4 and 48 h post administration, the mice were or were not irradiated with US (1.0 MHz, 2.0 W/cm^2^, 50 duty cycle) for 5 min. The ultrasonic coupling agents were replaced 2 times during the period to prevent the thermal effect of the US. The body weights and tumor sizes were measured every 2 d for consecutive 16 days. The tumor volume was calculated by the following equation: V= (tumor length)×(tumor width)^2^. At the end of the treatment, the mice were sacrificed and their tumors and major organs were harvested and fixed with 4 % paraformaldehyde for H&E staining. Tumor sections were further stained with TUNEL and Ki67 according to the manufacture’s instruction. Finally, above sections were imaged by a fluorescence microscope.

### In vivo evaluation of ICD effects induced by SDT

The subcutaneous CT26 tumor-bearing mice were received the intratumor injection of PBS and TIR@siRNA and then some mice were exposed to US irradiation at 4 h post administration as above experiments mentioned. After further raised for 12 h, the tumor tissues of the mice were harvested and processed into 5-µm cyro-sections. Then the sections were stained with FITC-conjugated anti-CRT primary antibody (1:100) and anti-HSP70 primary antibody (1:100) followed staining with PE-conjugated secondary antibody (1:200) by the immunofluorescence staining technique. Besides, at 24 h post treatments, the tumor tissues were made into 5-µm paraffin sections and processed staining with anti-CD11c primary antibody (1:100) and PE-conjugated secondary antibody (1:200). Then the sections were imaged by a fluorescence microscope to evaluate SDT induced ICD effect and recruitment of DC cells in tumor tissues.

### Anti-metastasis effect of SDT combined anti-PD-L1 therapy in vivo

Orthotopic colorectal metastatic tumor models were firstly implanted on 6 d post the inoculation of subcutaneous CT26 tumor. T hen the mice were randomly divided into 4 treatment groups (n = 6): (1) PBS as control; (2) ^D^PPA-1 peptide; (3) TIR@siRNA with US irradiation; (4) TIR@siRNA with US irradiation plus ^D^PPA-1 peptide. The TIR@siRNA were intratumorally injected on day 0 and day 4 at IR780 dosage of 2.0 mg/kg and Nrf2-siRNA dosage of 0.5 mg/kg. The US irradiation was given at 4 and 48 h post administration as the same condition as above treatment applied. The ^D^PPA-1 peptide was intravenously injected on day 0, 2, 4, 6, 8, 10 at the dosage of 40 mg/kg. The body weights and subcutaneous tumor sizes were measured every 2 days for consecutive 16 days. On day 4, three mice of each group was sacrificed and the part of subcutaneous tumor tissues were harvested to be processed as paraffin sections. The CD4^+^ and CD8^+^ T cells in the tumor tissues were stained with anti-CD4, anti-CD8 primary antibody (1:100) and PE-conjugated, FITC-conjugated secondary antibody (1:200) by the immunofluorescence staining technique, respectively. Meanwhile, the IFN-γ and TNF-α expressed in CD4^+^ and CD8^+^ T cells were also evaluated by the Immunofluorescence histochemical analysis to assess the immune activation. At the end of treatments, the subcutaneous tumor and the whole intestines were collected and imaged by a mobile phone. The metastatic nodules on the intestines of every mouse were counted for statistical analysis. Besides, the spleen tissues were collected for the CD4^+^ and CD8^+^ T cells analysis as the same protocol as above mentioned. The fluorescence semi-quantitative analysis was conducted through the software of Image Pro Plus. In addition, the expressions of CD44 and CD62L on T cells in spleen tissues were also detected by the flowcytometry to evaluate the specific antitumor memory effect of these treatments. The above treatment experiment was repeatedly conducted for once.

### Statistical analysis

All data were presented as the mean ± standard deviation (SD) of at least three independent experiments. Statistical differences were determined by the Student’s t test for comparison of 2 groups and one-way ANOVA for multiple groups. Values of *P* < 0.05 were considered statistically significant.

## Results and discussion

### Synthesis, preparation and characterization of the TIR@siRNA nanoparticles

The dye IR780, with excellent NIR fluorescence properties, has been promisingly applied as a sonosensitizer in SDT in previous studies [34, 35]. Due to its strong lipophilicity and lack of modifiable groups, the biomedical applications of IR780 are limited to a great extent. Numerous studies have improved the hydrophilicity of IR780 by using nanomaterials (e.g., micelles, mesoporous silica and liposomes) through physical encapsulation. Here, we used a chemically coupled approach to improve the hydrophilicity of IR780 for application in SDT against cancer. The TAT peptide with better water solubility reacted with IR780 through a substitution reaction, and the chemical structure of the product TAT-IR780 is shown in Scheme 1A. Thin-layer chromatography was used to confirm the formation of the new substances. The chromatographic behaviors of the product, IR780 and TAT peptide mixed with IR780 were completely different, as observed from Additional file 1: Fig. S1A. Furthermore, the molecular weight of TAT-IR780 was characterized by using mass spectrometry and calculated to be 2165 from the peaks of the tetravalent, trivalent and bivalent ions observed at m/z 541.8, 722.6 and 1083.7, respectively (Additional file 1: Fig. S1B). The calculated molecular weight of TAT-IR780 was consistent with the value given by ChemDraw software, demonstrating the successful synthesis of TAT-IR780.

**Fig. 1:**
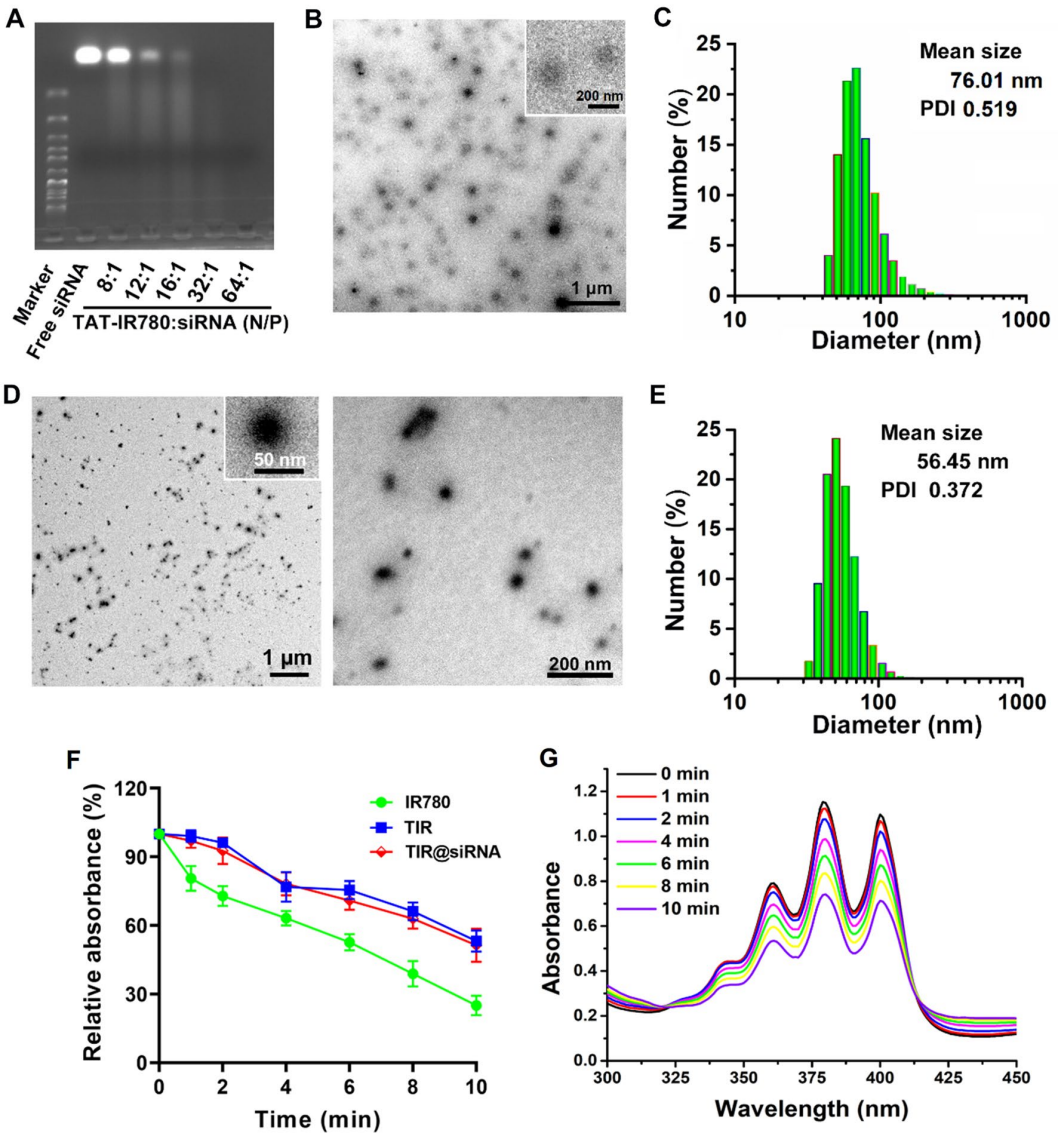
Characterization and sonodynamic effficiency of TIR@siRNA. **A** Agarose gel electrophoresis image of the mixture of TAT-IR780 and Nrf2-siRNA at different N/P ratios. TEM image **B** and size, size distribution **C** of TIR nanoparticles. TEM image **D** and size, size distribution **E** of TIR@siRNA nanoparticles. The inset in **B** and **D** are the high-resolution TEM images. **F** The UV-Vis absorption changes of IR780, TIR and TIR@siRNA under US irradiation for different times. **G** The UV-Vis spectra of ABDA with the TIR@siRNA at different time interval of US irradiation

Next, we assessed the gene loading capacity of TAT-IR780 to load Nrf2-siRNA through a gel retardation assay due to the high positive charge of the TAT peptide. As shown in Fig. 1A, the migration of Nrf2-siRNA was completely inhibited when the N/P ratio was increased to 32/1. Thus, the zeta potentials of TIR and the nanocomplexes with N/P ratios of 32/1 and 64/1 were + 34.2 mV, + 11.9 mV and + 22.5 mV, respectively. Considering the zeta potential, in an effort to make the prepared particles more compact and smaller to meet the need for nuclear targeting and safeguard the full loading of siRNA, the high N/P ratio of 64/1 was chosen to prepare the nuclear-targeting system. The TEM images showed that TIR was around 200 nm with low contrast while the hydration diameter of TIR was 76.01 nm with a PDI of 0.519 (Fig. 1B, C), which might due to the weak assembly force of TIR resulting in the morph during the drying process. After co-assembled with siRNA, the morphology of the prepared TIR@siRNA nanoparticles showed a solid nanosphere structure with a diameter of approximately 50 nm (Fig. 1D). The average hydrodynamic diameter of TIR@siRNA detected by dynamic light scattering (DLS) was 56.45 nm with a PDI of 0.372 (Fig. 1E). Notably, there is a large overlap between the particle size distribution range of TIR@siRNA and the diameter of the nuclear pore complex (20-70 nm) [52], which indicates that TIR@siRNA could enter the nucleus to a great extent.

Chemical modification and physical encapsulation have been demonstrated to improve the optical stability of NIR dyes [53, 54]. Hence, the changes in the UV–Vis and fluorescence spectra of IR780, TIR, and TIR@siRNA during 5 days of storage in water were evaluated and are shown in Additional file 1: Fig. S2. Compared with IR780, the UV-vis absorption decreased more slowly and to a lesser extent in both the TIR and TIR@siRNA samples (Additional file 1: Fig. S2A–C). Moreover, as shown in Additional file 1: Fig. S2D, the same phenomenon was observed by the fluorescence decay results. Excitingly, the optical stability was improved to a certain extent after Nrf2-siRNA adsorption, which could be ascribed to the self-assembly mediated by the positive and negative charges of TIR and the siRNA further protecting IR780.

### In vitro ultrasonic stability and sonodynamic efficiency of TIR@siRNA

A previous study reported that anthocyanin photosensitizers are photobleached during laser irradiation with UV-Vis attenuation, which restrains their PDT effects [55]. Here, the UV-Vis absorbance at 785 nm of IR780, TIR and TIR@siRNA under US irradiation (1.0 W/cm^2^, 50% duty cycle) for 10 min was measured to evaluate the ultrasonic stability. From Fig. 1 F, enhanced stability was observed after the US irradiation of IR780 after TAT peptide modification. Next, we evaluated the SDT efficiency of TIR@siRNA upon US irradiation by measuring ROS generation using ABDA as an indicator. As displayed in Fig. 1G, ABDA in the TIR@siRNA nanoparticles exhibited visible changes in its UV absorption spectrum, showing a gradual decline during the 10 min of US irradiation. Based on previous research [48], this might be attributed to the decomposition of ABDA by the ROS generated from TIR@siRNA under US irradiation. Considering the results above, we proposed that TIR@siRNA could be used as a SDT sonosensitizer for colorectal cancer therapy.

### Cellular uptake and nuclear localization of the TIR@siRNA nanoparticles

**Fig. 2:**
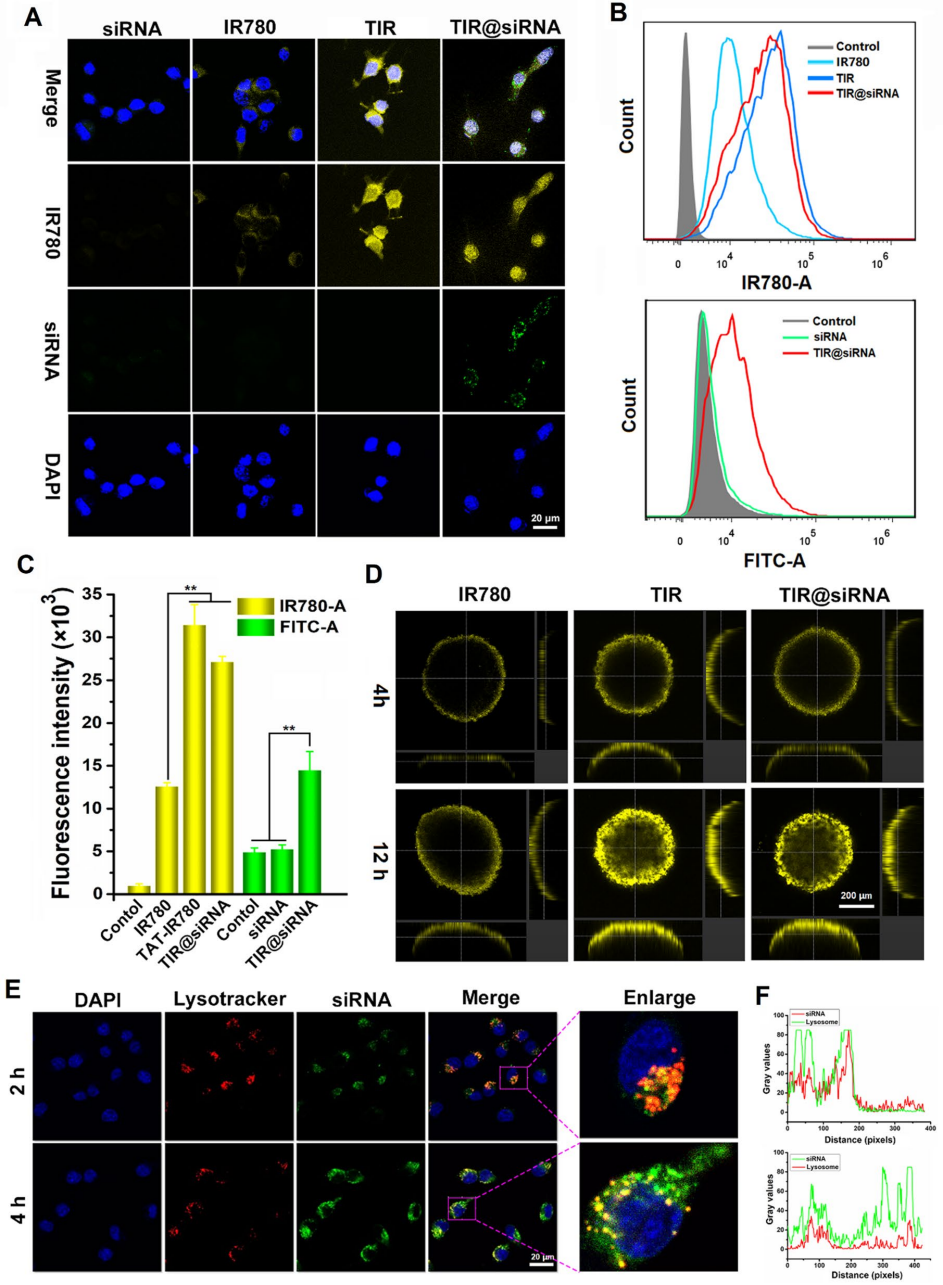
**A** Confocal images, **B** flow cytometry curves and **C** quantitative analysis of CT26 cells after 4 h incubation with FITC-Nrf2-siRNA, IR780, TIR and TIR@FITC-Nrf2-siRNA. **D** Confocal images of CT26 tumor spheroids after 4 and 12 h incubation with IR780, TIR and TIR@siRNA. **E** Confocal images of CT26 cells after 2 and 4 h incubation with TIR@siRNA while the nuclei stained with DAPI and lysosome stained with lysotracker red. **F** The fluorescence colocalization curves of red and green fluorescence at 2 h (upper) and 4 h (downer). *P < 0.05 and **P < 0.01 for the comparisons between two treatment groups

Based on the transmembrane effect and nuclear targeting function of the cell-penetrating peptide, we first evaluated the cellular uptake and subcellular distribution of TIR@FITC-Nrf2-siRNA in CT26 colorectal cancer cells. The autofluorescence of both IR780 and FITC were used as observation targets of IR780 and Nrf2-siRNA. Figure 2A shows confocal images of CT26 cells after incubation with FITC-Nrf2-siRNA, free IR780, TIR and TIR@FITC-Nrf2-siRNA for 4 h. Compared to the scattered distribution of IR780 in the cytoplasm in the free IR780 treatment group, IR780 fluorescence in the TIR treatment group was mainly observed in the perinuclear region, and some IR780 entered the nucleus. Surprisingly, IR780 was mostly located in the nucleus in the TIR@FITC-Nrf2-siRNA treatment group, which might be attributed to the small diameter of the TIR@siRNA nanoparticles formed by the self-assembly of the strong charge interaction, which is close to the size of the nuclear pore complex (~70 nm) [52]. Furthermore, stronger fluorescence intensity from IR780 was observed in both the TIR and TIR@FITC-Nrf2-siRNA treatment groups. Compared to the faint green fluorescence in the FITC-Nrf2-siRNA treatment group, much stronger FITC fluorescence was observed in the TIR@FITC-Nrf2-siRNA group. For the green fluorescence mainly distributed in cytoplasm, this might due to the gene release in the acidic lysosomes. The above results suggested that TIR@siRNA could efficiently deliver IR780 and Nrf2-siRNA into CT26 cells and improve the nuclear distribution of IR780 to the exert gene-facilitated nuclear-targeting SDT. In addition, the fluorescence intensities of IR780 and FITC were quantitatively measured by flow cytometry, and the results are shown in Fig. 2B, C. Quantitative analysis indicated that the fluorescence intensity of IR780 in TIR@FITC-Nrf2-siRNA- and TIR-treated cells was 2.2-fold and 2.5-fold higher than that in the free IR780 treatment group, respectively, and 2.8-fold more Nrf2-siRNA was delivered into CT26 cells by TIR@siRNA than free Nrf2-siRNA. This further demonstrated the improved cellular internalization and gene loading capacity of the TAT peptide.

### Penetration of the TIR@siRNA nanoparticles into colorectal tumor-like spheroids

A previous report revealed that the cell-penetrating peptide TAT can mediate the penetration of nanoparticles into deep tumors [56]. Therefore, we constructed a 3D colorectal tumor cell sphere to investigate the distribution of TIR@siRNA. As shown in Fig. 2D, more IR780 was distributed over a wider range in the TIR- and TIR@siRNA-treated CT26 tumor spheres at both 4 and 12 h. With the extension of the incubation time, the fluorescence intensity was further enhanced. However, an insignificant enhancement was observed in the IR780 treatment group, which might be attributed to the high hydrophobicity of IR780. The slightly weaker fluorescence intensity of IR780 in the TIR@siRNA group than the TIR treatment group might be due to the penetration of the TAT peptide, which depends on the positive charge.

### Evaluation of the lysosomal permeabilization and lysosomal escape behavior of the TIR@siRNA nanoparticles

As previously shown, the highly positive charge of the TAT peptide-modified nanoparticles could destabilize lysosomal membranes, thus promoting lysosomal escape [40]. To assess the lysosomal permeabilization of the TIR@siRNA nanoparticles, cells after various treatments were stained with acridine orange (AO) and then analyzed by confocal microscopy and flow cytometry. AO is a fluorescent dye whose fluorescence changes depending on the pH, emitting a red signal at the acidic pH of the lysosomes and a green signal at the neutral pH of the cytosol and nucleus [57]. As expected, AO showed a red perinuclear spotted signal and weak green cytosolic fluorescence in PBS- and IR780-treated CT26 cells, while upon treatment with TIR and TIR@siRNA, the nuclei produced more intense green signals (Additional file 1: Fig. S4A), indicative of moderate lysosome basification by the TAT peptide. In addition, the decrease in red fluorescence and increase in green fluorescence were demonstrated by flow cytometry (Additional file 1: Fig. S4B, C). The above results revealed that TIR and TIR@siRNA could enhance lysosomal membrane permeability.

As TIR@siRNA changes lysosomal permeability, we next evaluated the lysosomal escape behavior of Nrf2-siRNA. FITC-conjugated Nrf2-siRNA was used to observe the colocalization of Nrf2-siRNA and lysosomes, which were stained with LysoTracker red. From Fig. 2E, we can clearly observe that Nrf2-siRNA was mostly located in the lysosomes after CT26 cells were incubated with TIR@ FITC-Nrf2-siRNA for 2 h, while Nrf2-siRNA was mostly distributed in the cytoplasm after 4 h with increased cell internalization. The fluorescence colocalization curves (Fig. 2F) also showed that the Nrf2-siRNA from TIR@siRNA could escape from the lysosomes and distribute into the cytoplasm, thus playing a role in inhibiting tumor cell redox balance regulation after SDT.

### In vitro antitumor effects of gene-augmented nuclear-targeting SDT against colorectal cancer

**Fig. 3:**
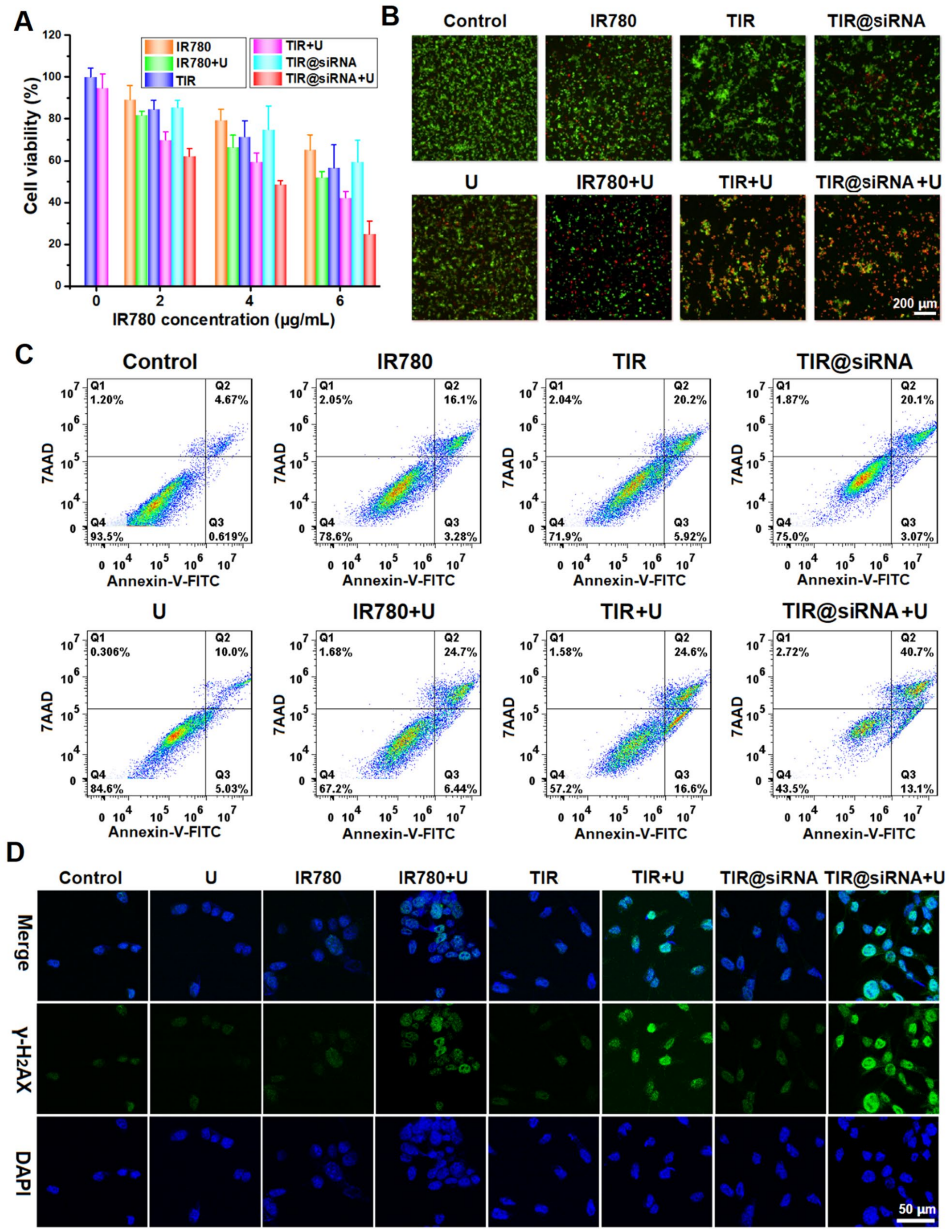
In vitro antitumor effect on colorectal CT26 cells of TIR@siRNA mediated gene enhanced nuclear-targeting SDT. **A** Cytotoxicities on CT26 cells after 24 h treatments of free IR780, TIR and TIR@siRNA with/without US irradiation. **B** The LIVE/DEAD analysis by confocal microscope and **C** the cell apoptosis analysis by flow cytometry of CT26 cells at 24 h after various treatments (Q1: dead cells; Q2: late apoptotic cells; Q3: early apoptotic cells; Q4: live cells). **D** Confocal images of CT26 cells stained with DAPI and antibodies against γ-H2AX after various treatments. US irradiation was carried out at a power density of 1.0 W/cm^2^ for 5 min with 50% duty cycle

The in vitro antitumor effects of TIR@siRNA combined with US irradiation was first evaluated in CT26 cells by MTT assay, and the results are outlined in Fig. 3A. Free IR780, TIR and TIR@siRNA without US irradiation all exhibited certain cytotoxicities at various concentrations, which might be ascribed to the membrane disruption by the positively charged IR780. After the administration of US irradiation, the cytotoxicities of the above treatments were notably enhanced, demonstrating the killing effects of SDT on CT26 cells. In addition, compared to free IR780, the cytotoxicities of TIR and TIR@siRNA combined with US irradiation were further enhanced, which showed the superiority of nuclear-targeting SDT. Furthermore, combined with the redox regulation based on Nrf2-siRNA, TIR@siRNA-mediated SDT more effectively killed CT26 cells at various concentrations. Incidentally, US irradiation only caused 5% inhibition of cell viability, which illustrates the superior adaptability of SDT.

To visibly identify live and dead cells, a live/dead kit was applied for cell staining after various treatments, and fluorescence microscopy was adopted to produce images in which green and red refer to live and dead cells, respectively. As shown in Fig. 3B, free IR780, TIR and TIR@siRNA combined with US irradiation killed CT26 cells to different degrees, but TIR@siRNA with US irradiation exhibited the strongest cell-killing efficacy. Next, we conducted an apoptosis assay in CT26 cells after various treatments by using annexin-V-FITC and 7-AAD staining. Figure 3C shows the corresponding flow cytometric analysis. Moderate induction of apoptosis was observed in CT26 cells after 24 h of treatment with free IR780, TIR and TIR@siRNA at an IR780 concentration of 4 µg/mL. Clearly, the cells subjected to US irradiation showed notably increased apoptosis rates, revealing the activated SDT effects of IR780 on CT26 cells. The apoptosis rates after treatment with free IR780, TIR and TIR@siRNA combined with US irradiation were 33.8%, 43.8% and 56.5%, respectively. The above results prove that TAT peptide-mediated nuclear-targeting SDT exhibits a higher killing effect on CT26 cells, and the cytotoxicity is further enhanced with the gene silencing effects of Nrf2-siRNA.

### Gene-augmented SDT effects and the mechanism of TIR@siRNA against colorectal cancer cells

According to a previous report [58], intranuclear photodynamic therapy (PDT) can more directly and efficiently kill cancer cells by causing irreversible damage to double-stranded DNA. The mechanism of SDT against cancer is the same as that of PDT; thus, we evaluated DNA damage after SDT. H2AX phosphorylation at Ser139 (γ-H2AX) is one of the earliest events to occur in response to DNA damage [59]. As TIR@siRNA can deliver IR780 into the nucleus after cell uptake, we evaluated the expression level of γ-H2AX in CT26 cells after various treatments by immunofluorescence. As outlined in Fig. 3D, the phosphorylation level of γ-H2AX clearly increased after US irradiation, indicating that SDT induced DNA damage. Excitingly, treatment with TIR and TIR@siRNA in combination with US irradiation showed increased DNA damage, further demonstrating the superiority of nuclear-targeting SDT against cancer cells.

**Fig. 4:**
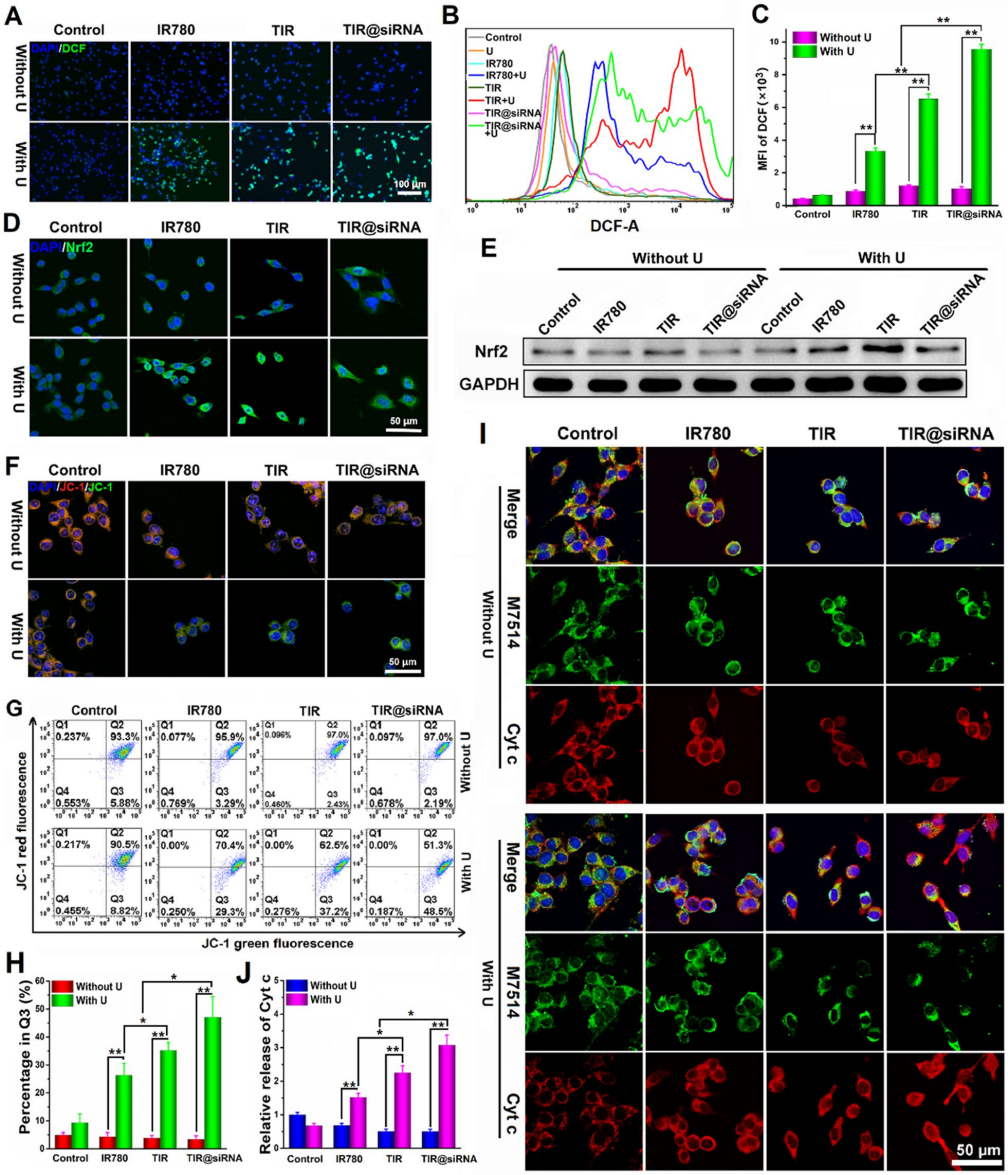
in vitro SDT efficiencies of TIR@siRNA in CT26 cells. Fluorescence images (**A**), flow cytometry curves (**B**) and quantitative analysis (**C**) of the generations of intracellular ROS after various treatments. Confocal images (**D**) and western blot analysis (**E**) of expressions of Nrf2 of various treatments. Confocal images (**F**) and flow cytometry analysis (**G**) and quantitative analysis in Q3 quadrant (**H**) of the membrane potential changes of mitochondrial stained with JC-1 after various treatments. **I** Confocal images of the cells after 24-hour treatments of free IR780, TIR and TIR@siRNA with or without US irradiation. Mitochondria and Cyt c stained respectively with MitoTracker green (M7514) and anti-Cyt c antibody emitted green and red fluorescence. **J** Relative released Cyt c from mitochondrial to cytoplasm (the control was set as 1). *P < 0.05 and **P < 0.01 for the comparisons between two treatment groups

The antitumor effects of SDT are primarily based on the ROS generated by the sonosensitizer under US irradiation. DCFH-DA, a fluorescence indicator of ROS, was used to evaluate the ROS levels in CT26 cells after various treatments. As shown in Fig. 4A, CT26 cells treated with free IR780, TIR and TIR@siRNA combined with US irradiation exhibited more intense green fluorescence compared to the treatments without US irradiation, indicating the large amounts of ROS generated by SDT. Additionally, we quantitatively assessed the intracellular ROS levels by flow cytometry and acquired similar results to those above (Fig. 4B, C). Compared to free IR780, TIR and TIR@siRNA with US irradiation remarkably increased the intracellular ROS level. In addition, TIR@siRNA with US irradiation treatment showed a higher ROS level than TIR treatment after US irradiation, proving that the gene silencing effect of Nrf2-siRNA could inhibit the ROS depletion initiated by Nrf2. We then evaluated the intracellular expression levels of Nrf2 12 h after SDT. From Fig. 4D, E, upregulation of the Nrf2 protein was clearly observed after SDT and IR780, TIR and TIR@siRNA treatment, demonstrating that the increased ROS activated the redox regulatory pathway based on Nrf2 [48]. Moreover, higher Nrf2 expression was observed in the TIR group than in the free IR780 group (both after US irradiation) in response to the higher ROS levels induced by SDT based on TIR. Excitingly, Nrf2 expression in the TIR@siRNA with US irradiation treatment group was dramatically lower than that in the free IR780 and TIR SDT groups, revealing that TIR@siRNA could block the Nrf2-based ROS depletion pathway to enhance the antitumor effects of SDT.

As ROS can oxidize the mitochondrial membrane to block the respiration chain [60], cellular mitochondrial membrane potential changes were evaluated with a JC-1 assay. JC-1 is a fluorescent probe which displays different fluorescence in response to mitochondrial membrane electric potential change with red (JC-1 aggregates) of high potential for normal mitochondrial and green (JC-1 monomers) of low potential for damaged mitochondrial. As shown in Fig. 4F, the majority of the mitochondria displayed red fluorescent dots in the control, IR780, TIR, TIR@siRNA and US irradiation only groups, confirming the normal mitochondrial membrane potential. Upon US irradiation, the red fluorescence of the mitochondria in all SDT groups disappeared and the green fluorescence was enhanced, which corroborated the enhanced decrease in mitochondrial membrane potential by the ROS generated by SDT. Quantitative analysis by flow cytometry (Fig. 4G) showed that SDT after treatment with IR780, TIR and TIR@siRNA caused 23.42%, 31.32% and 42.62% JC-1 aggregate disappearance, which indicated that more mitochondria were damaged by the enhanced SDT through both the increased endocytosis mediated by TAT and the blocked regulation of redox balance by Nrf2. The percent of cells got involved in Q3 quadrant (the cells whose mitochondrial were damaged) was correspondingly shown in Fig. 4 H.

A large amount of ROS causing damage to the mitochondrial membrane triggers the release of Cyt c from mitochondria into the cytoplasm to activate the mitochondria-mediated apoptosis pathway, which is considered to be the main mechanism of SDT against cancer [61]. Subsequently, we observed the subcellular colocalization between Cyt c and mitochondria in CT26 cells to assess Cyt c release after various treatments by immunofluorescence. Figure 4I shows the fluorescence images of these treated cells and the relative amount of released Cyt c was analyzed via Image Pro Plus software (Fig. 4J). Notably, the red fluorescence from Cyt c and green fluorescence from the mitochondria almost completely overlapped in the control group and other groups without US irradiation. Upon exposure to US irradiation, free IR780, TIR and TIR@siRNA treatment all induced Cyt c release to varying degrees. Moreover, TIR@siRNA with US irradiation caused Cyt c release into the cytoplasm to the greatest extent, which should be ascribed to more IR780 being delivered into CT26 cells through the TAT peptide and the gene silencing effects of Nrf2-siRNA. The above results certified that TIR@siRNA possessed strong SDT efficacy and efficiently activated the mitochondrial apoptosis pathway in colorectal cancer cells after US irradiation.

### In vivo retention and biodistribution of TIR@siRNA

**Fig. 5:**
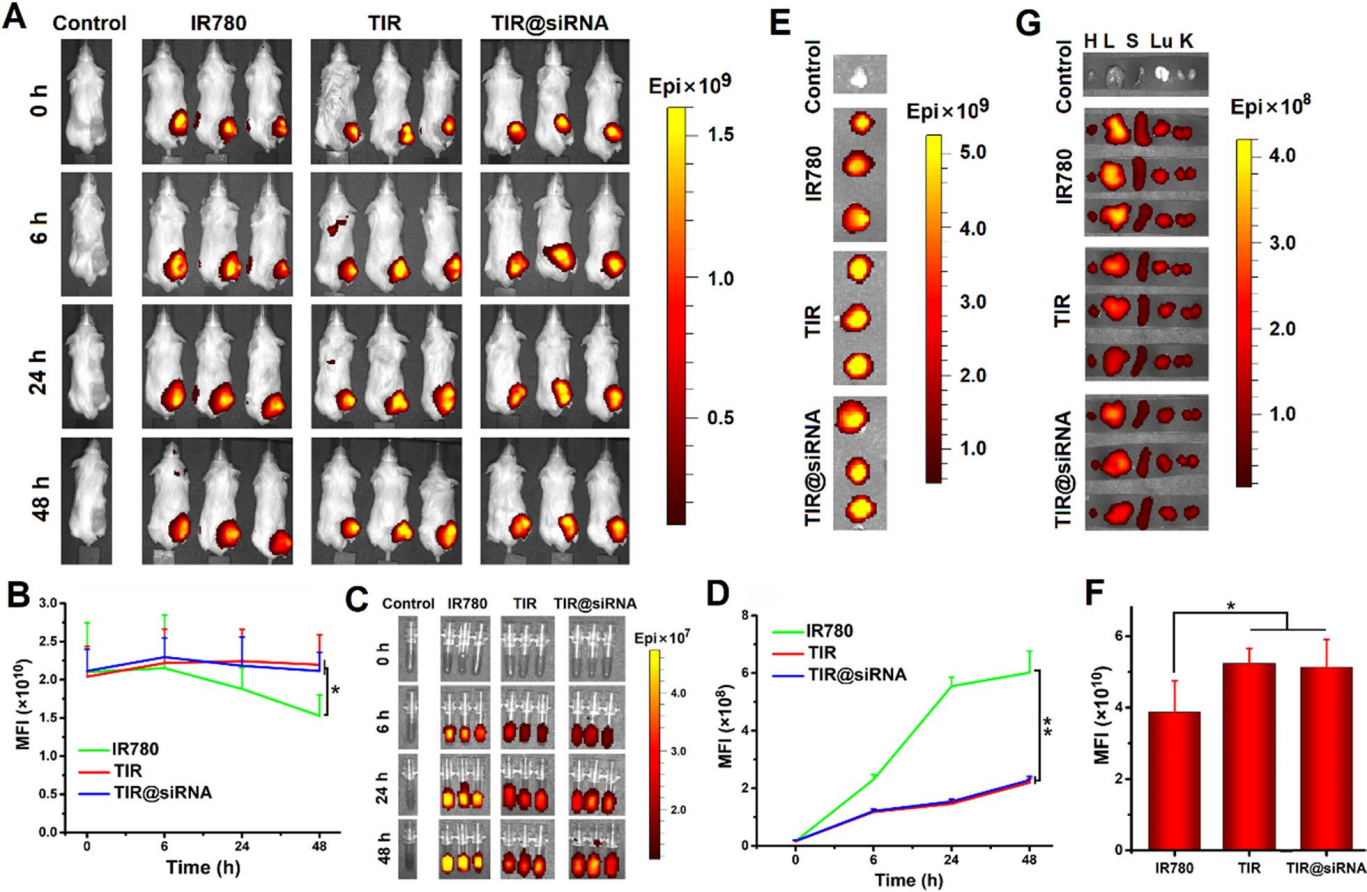
In vivo tumor retention and tissue distribution of TIR@siRNA in CT26 tumor bearing mice. **A** In vivo fluorescence images of the mice and **B** the change curves of mean fluorescence intensity of tumors at 0 h, 6 h, 24 and 48 h after intratumoral injection of normal saline, free IR780, TIR and TIR@siRNA. **C** Fluorescence images and **D** mean fluorescence intensity changes of diluted whole blood derived from the mice at the corresponding time points. Fluorescence images (**E**) and mean fluorescence intensity (**F**) of ex vivo tumor tissues and major organs (**G**) of the mice at 48 h post administration. All data are presented as the mean± SD (n = 3). *P < 0.05 and **P < 0.01 for the comparisons between two treatment groups

As previously reported, modifying nanoparticles with the TAT peptide can enhance their adhesion to tumor tissues, preventing removal by blood flow [62]. Thus, an in vivo imaging system was used to detect the fluorescence of IR780 that remained in tumor tissues and was transported into the blood and in several major organs at different times 48 h after intratumoral injection of free IR780, TIR and TIR@siRNA. Fluorescence images of mice and blood at different time points after administration of normal saline, free IR780, TIR and TIR@siRNA are shown in Fig. 5A, C and the quantitative fluorescence intensity changes are severally displayed in Fig. 5B, D. The contents of free IR780 evidently decreased in tumor tissues and entered the blood over time. By comparison, TIR and TIR@siRNA showed slight attenuation of IR780 in the tumor tissues and little entry into the blood. Next, the mice were sacrificed after 48 h, and the tumors and major organs were collected for further distribution assessments. As outlined in Fig. 5E–G, the fluorescence of IR780 in the TIR- and TIR@siRNA-treated tumors was notably stronger than that of free IR780 and weaker in the major organs. The above results demonstrated that TIR and TIR@siRNA could deter drug entry into the blood and drug distribution into the major organs, thus enhancing tumor retention compared to free IR780. It was therefore determined that TAT decoration could keep the majority of the drug in the tumor tissues for a long time, reducing the frequency of administration needed for further treatment.

### In vitro and in vivo photoacoustic imaging

Based on the photothermal effects of IR780, which can generate PA signals under laser irradiation, we then evaluated the PA imaging abilities of TIR@siRNA. As shown in Additional file 1: Fig. S5A, the PA signal intensity of TIR@siRNA and IR780 both displayed the concentration dependence and no obvious difference between TIR@siRNA and IR780. When the concentration of IR780 in TIR@siRNA reached 50 µg/mL, strong PA signals were clearly observed. Additional file 1: Fig. S5B shows the in vivo PA imaging of TIR@siRNA and IR780. The tumors treated with TIR@siRNA and IR780 both showed strong PA signals at 0.5 h post intratumoral injection which suggested the great self-monitring performance of TIR@siRNA. As the time going on, the PA signals in tumor tissues gradually attenuated, which could be attributed to the drug diffusion induced concentration decrease and the metabolism of drugs from the tumor site. Meanwhile, the PA signals in TIR@siRNA treated tumors are always stronger than which treated with IR780 at each time points due to the prolonged tumor retention of TIR@siRNA. The above results demonstrated that TIR@siRNA has potential as a PA imaging agent for monitoring drug contents in tumor tissues.

### In vivo gene-augmented SDT efficacy of TIR@siRNA

**Fig. 6:**
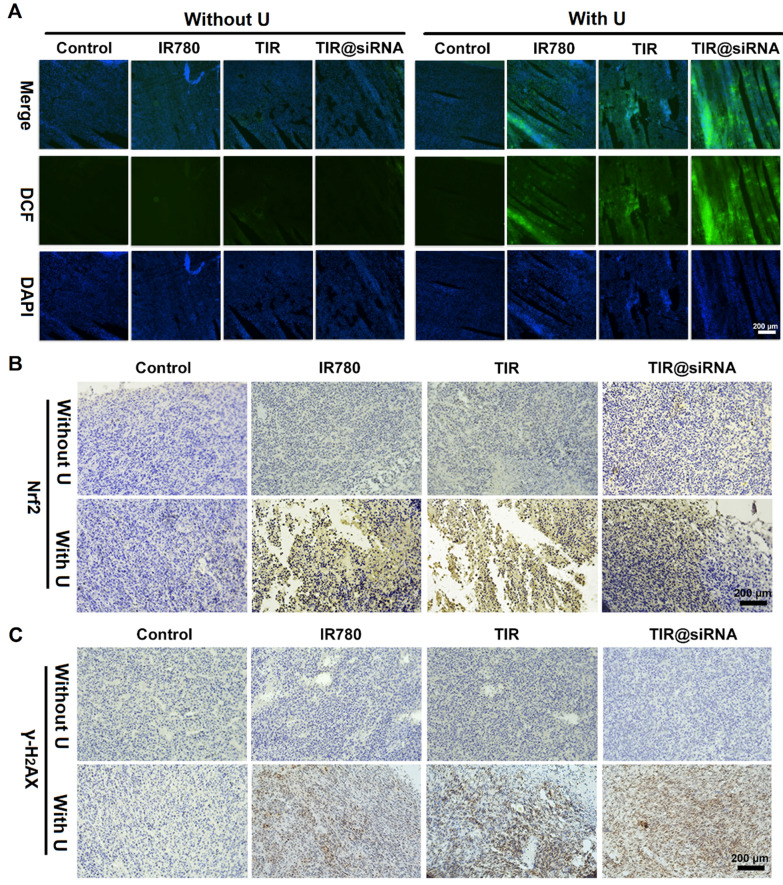
in vivo SDT efficiency of TIR@siRNA in CT26 tumor-bearing mice. **A** Fluorescence images of tumor sections processed with SOSG at 12 h after various treatments. Microscope images of tumor sections with immunohistochemical staining of **B** Nrf2 and **C** γ-H2AX at 12 h after various treatments. In all above experiments, the doses of IR780 and Nrf2-siRNA were 2.0 and 0.5 mg/kg and the US irradiation conducted at the power of 2.0 W/cm^2^ for 5 min (1 MHz, 50% duty cycle)

To evaluate the SDT efficacy of TIR@siRNA in vivo, the intratumoral generation of ROS was evaluated by using the fluorescent probe SOSG. Figure 6A shows the fluorescence images of the mouse tumor sections after various treatments for 12 h. After US irradiation, strong green fluorescence intensity was visibly observed in the tumor tissues treated with free IR780, TIR and TIR@siRNA, indicating that these treatments induced the generation of ROS and exhibited potent SDT efficacy. In particular, TIR@siRNA with US irradiation exhibited notably higher intratumoral ROS than free IR780 and TIR, both with US irradiation, which contributed to the Nrf2-siRNA-mediated gene silencing effect. Furthermore, we evaluated Nrf2 expression by immunohistochemical staining, and the microscopic images are shown in Fig. 6B. Clearly, the tumor tissues treated with IR780 and TIR combined with US irradiation exhibited higher Nrf2 expression than those without US irradiation or US irradiation alone, which revealed that the ROS-induced imbalance in redox homeostasis activated the Nrf2-based antioxidant pathway. However, TIR@siRNA greatly decreased Nrf2 expression in tumor tissues after SDT compared to the free IR780- or TIR-treated SDT groups, suggesting that Nrf2-siRNA-induced gene silencing efficiently relieved ROS depletion after SDT. Next, we assessed whether the Nrf2-siRNA-mediated decrease in ROS production could counteract the DNA damage triggered by TAT-mediated nuclear targeting by evaluating γ-H2AX expression through immunohistochemical staining. As shown in Fig. 6C, all of the SDT treatment groups displayed clear increases in the expression of γ-H2AX, while TIR-based SDT exhibited much higher γ-H2AX expression than free IR780, which might be attributed to the TAT-based nuclear targeting. In addition, TIR@siRNA and US irradiation cotreatment further improved γ-H2AX expression, which revealed the superiority of gene silencing combined with nuclear-targeting SDT. These results further demonstrated that Nrf2-siRNA combined with nuclear-targeting SDT would become a synergistic sonosensitizer to amplify the anticancer efficacy of SDT by damaging double-stranded DNA.

### In vivo antitumor effects of TIR@siRNA with US irradiation

**Fig. 7:**
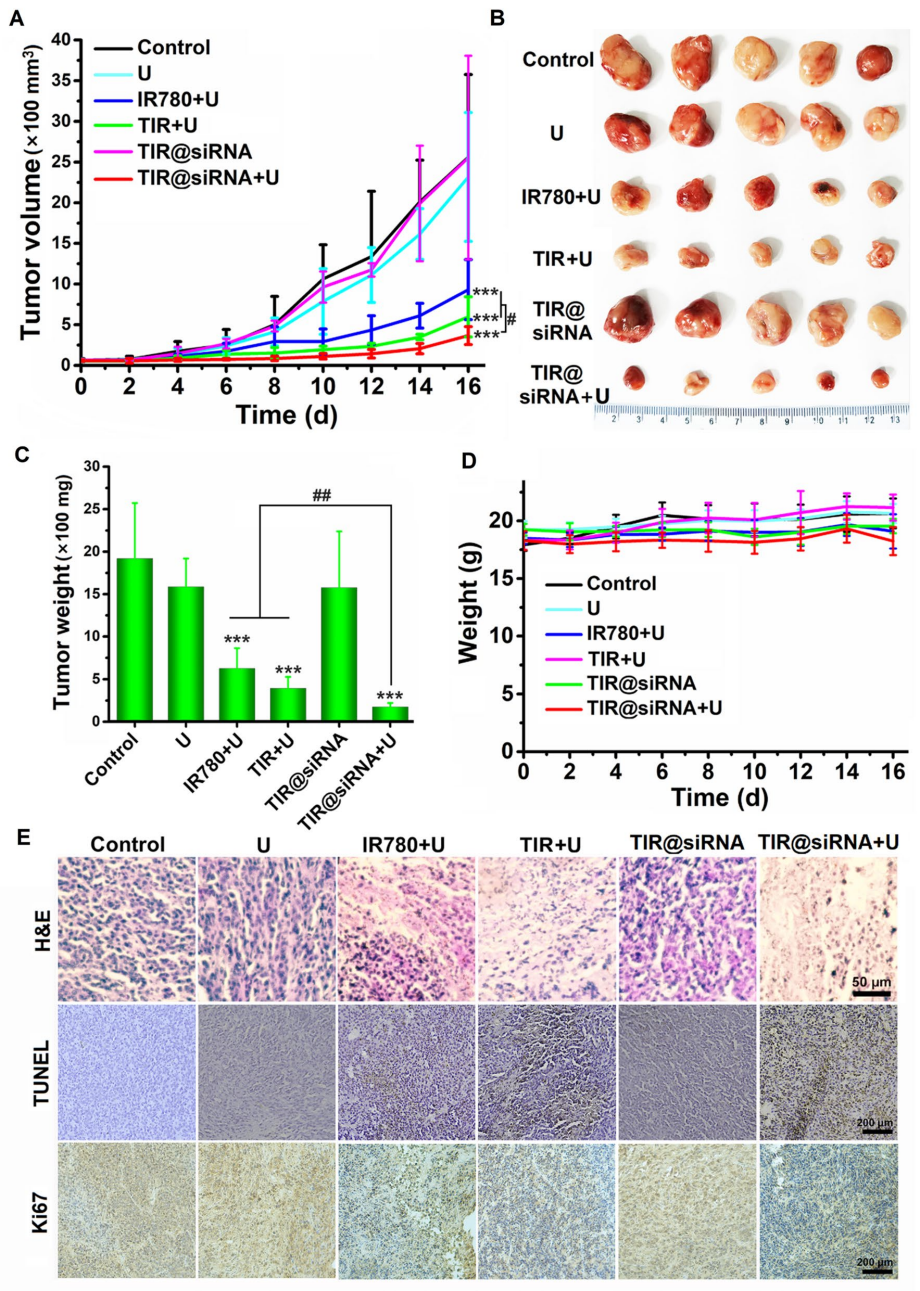
Anticancer effects of TIR@siRNA mediated gene augmented nuclear-targeting SDT in CT26 tumor-bearing mice. **A** Tumor growth curves during treatments of normal saline, US irradiation, TIR@siRNA and free IR780, TIR, TIR@siRNA combined with US irradiation (+U). Photos (**B**) and weights (**C**) of the tumors and dissected from the mice at the end of various treatments. **D** Body weights changes of the mice during various treatments. **E** Microscope images of tumor sections with H&E staining, TUNEL staining and immunohistochemical staining of Ki67. In above experiments, the doses of IR780 and Nrf2-siRNA were 2.0 mg/kg and 0.5 mg/kg, respectively. All data are presented as the mean± SD (n = 5). ***P < 0.005 as compared to the control and ^#^*P* < 0.05, ^##^*P* < 0.01 for the comparison between two treatment groups

Given that TIR@siRNA mediated gene augmented nuclear-targeting SDT showed promising results as described above, we further investigated its antitumor efficacy in CT26 bearing mice. Figure 7A shows the tumor growth curves during the various treatments and Fig. 7B outlines the picture of tumors harvested from the mice after the various treatments. Clearly, TIR@siRNA had no inhibitory effect on tumor growth, showing the same effects as those of free IR780 and TIR, which was proven in our previous report [47]; thus, the efficacies of free IR780 and TIR on CT26 tumor growth were not tested here. When combined with US irradiation, IR780, TIR and TIR@siRNA remarkably suppressed tumor growth, while US irradiation only showed negligible inhibition, which demonstrated the strong SDT effects of IR780 against tumors. Moreover, after US irradiation, TIR exhibited a greater suppressive effect than free IR780 due to the nuclear-targeting efficiency and increased tumor retention from the TAT peptide. When further combined with the strategy of blocking redox balance regulation after SDT, TIR@siRNA with US irradiation exhibited an evidently enhanced inhibitory effect on tumor growth and the smallest tumors were found in this group. The tumor weights shown in Fig. 7C further confirmed these results. After US irradiation, the tumor weights in the free IR780, TIR and TIR@siRNA treatment groups were 32.70%, 20.45% and 9.11% that of the control group.

During the treatment period, the body weight changes of the mice were monitored, and the results are shown in Fig. 7D. During treatment, the body weights of the mice in each group remained essentially constant, which initially indicated the great safety of these treatments. Next, we further assessed the pathological changes in the major organs and tumors of the mice after various treatments by H&E staining. Additional file 1: Fig. S6 shows microscopic images of the main organs, and no obvious pathological changes were observed in these tissues, which further demonstrated the biosafety of these treatments. The histopathological examination of tumor tissues was shown in Fig. 7E. Clearly, the SDT treatments remarkably induced the nuclear fragmentation and tissue necrosis of tumors. And the TIR with US irradiation group exhibited larger area of tumor necrosis and more nuclear fragmentation compared to free IR780 with US irradiation which elucidated the superiority of nuclear-targeting SDT. When further combined with Nrf2-siRNA, the tumor tissues displayed the most tissue necrosis, nuclear fragmentation and some lighter coloration and vacuolization of cytoplasm which demonstrated nuclear-targeting SDT combined Nrf2 inhibition could ablate the CT26 tumors to the greatest extent. In addition, TUNEL and Ki67 staining of tumor tissues was conducted to investigate the mechanism of TIR@siRNA against colorectal cancer under US irradiation. The TUNEL staining can detect the large number of sticky 3’-OH ends produced by the DNA double-strand break or single-strand break. Ki67 is a marker of cell proliferation which expressed in S, G2, and M phases of apoptosis, but is absent in G0 phase. The Ki67 proliferation index is closely related to the differentiation, invasion, metastasis and prognosis of many tumors. As shown in Fig. 7E, TIR@siRNA-mediated SDT exhibited the most significant effect on the induction of cell apoptosis and the inhibition of cell proliferation compared with the other treatments, which might be ascribed to the DNA damage induced by the nuclear-targeting treatment. All the above results indicated that TIR@siRNA can become an excellent SDT agent for colorectal cancer treatment based on nuclear targeting and Nrf2-siRNA-amplified SDT efficacy.

### Synergistic antitumor effects of gene augmented nuclear-targeting SDT boost anti-PD-L1 therapy in colorectal tumor bearing mice

**Fig. 8:**
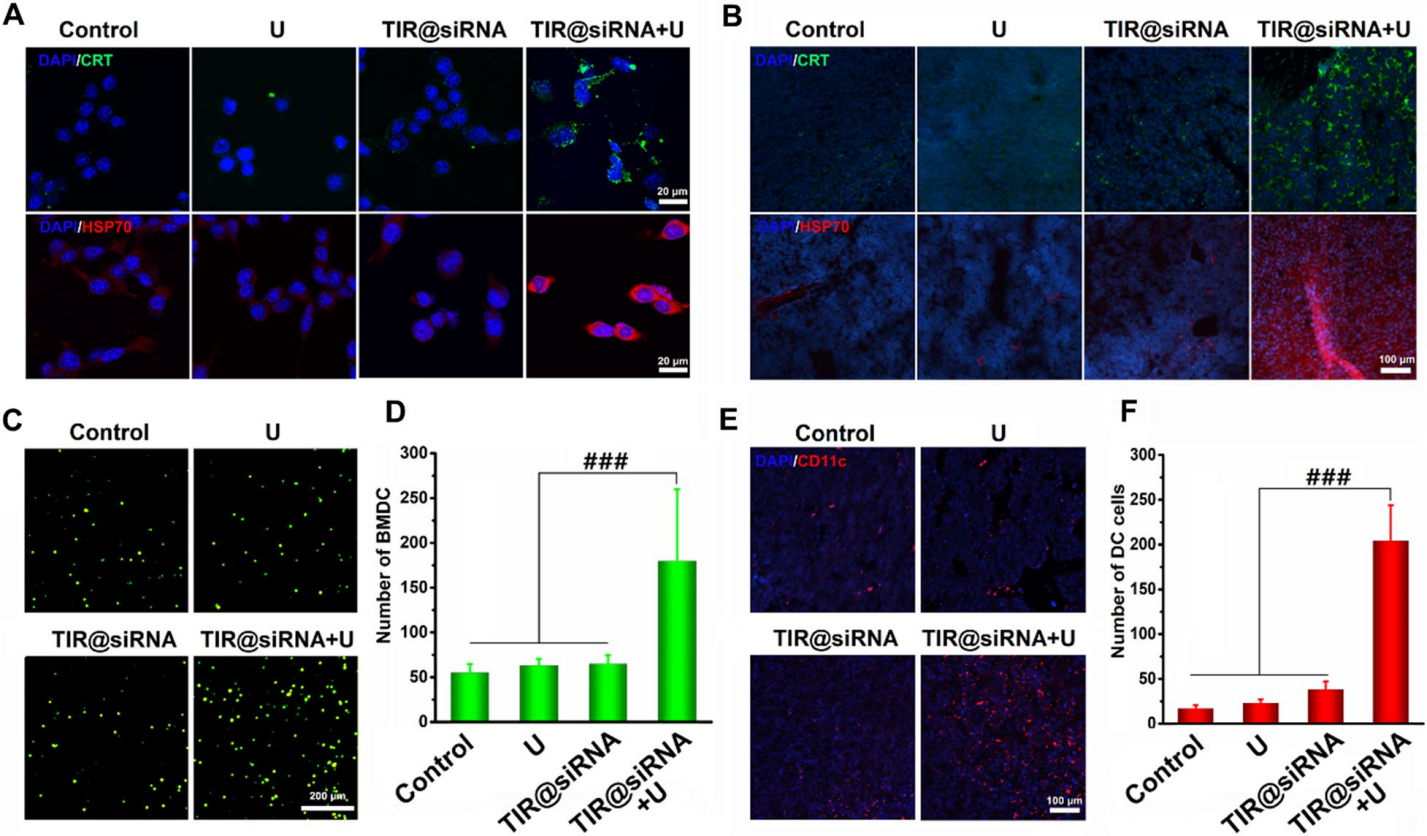
Evaluation of TIR@siRNA induced ICD effects and dentric cells recruitments in vitro and in vivo. Fluorescence images of calreticulin exposure and HSP70 expressions after various treatments in vitro (**A**) and in vivo (**B**). Fluorescence images (**C**) and quantitatively analysis (**D**) of BMDCs migrated into lower chamber of the transwell system by recruiting of the tumor cell lysates. Fluorescence images (**E**) and quantitatively analysis (**F**) of DC cells in tumor tissues at 24 h after various treatments. ^###^*P* < 0.005 for the comparison between two treatment groups

TIR@siRNA-mediated gene-augmented nuclear-targeting SDT evidently suppressed the growth of CT26 tumors but could not completely eliminate the tumor. Previous studies have revealed that SDT could significantly induce ICD in tumors to convert the tumors from a “cold” tumor into a “hot” tumor by remodeling the immunosuppressive tumor microenvironment to boost ICB therapy [27, 28]. Here, we assessed the synergistic antitumor effects of gene-augmented nuclear-targeting SDT combined with ^D^PPA-1 peptide-based anti-PD-L1 therapy on tumor ablation and metastasis. First, the ICD effects and DC recruitment induced by TIR@siRNA-mediated SDT in CT26 cancer cells were evaluated in vitro and in vivo. CRT exposure and HSP70 (a protein can be used for synergistic immunity) upregulation are the two major representative phenomena of ICD of cancer, then the immunofluorescence was contributed to assess the CRT exposure and HSP70 expression after the SDT [29]. As outlined in Fig. 8A, B, TIR@siRNA combined with US irradiation clearly induced CRT exposure and HSP70 upregulation in colorectal cancer in vitro and in vivo, which indicated the ICD in CT26 tumor after SDT. As the released tumor-associated antigens (TAAs) can attract DCs, cell lysates derived from the supernatants of the CT26 cells after various treatments were added to the lower chamber of a Transwell system to recruit BMDCs to the upper chamber. Figure 8C shows the images of calcein-AM-stained BMDCs migrating into the lower chamber, and Fig. 8D shows the corresponding quantitative analysis (4 fields of view were counted). Visibly, the TAAs released from the TIR@siRNA plus US irradiation-treated CT26 cells significantly recruited BMDCs compared to the cells treated in other ways. We further evaluated DC infiltration in CT26 tumor tissues 24 h after the various treatments. As shown in Fig. 8E, F, numerous DCs were recruited into CT26 tumor tissues after TIR@siRNA-based SDT in accordance with the in vitro assessment (4 fields of view were counted for quantitative analysis). These results suggested that TIR@siRNA-mediated gene-augmented nuclear-targeting SDT could induce ICD in colorectal cancer and recruit DCs into tumor tissues in vitro and in vivo for antigen presentation, thus improving the immunosuppressive microenvironment for ICB therapy.

**Fig. 9:**
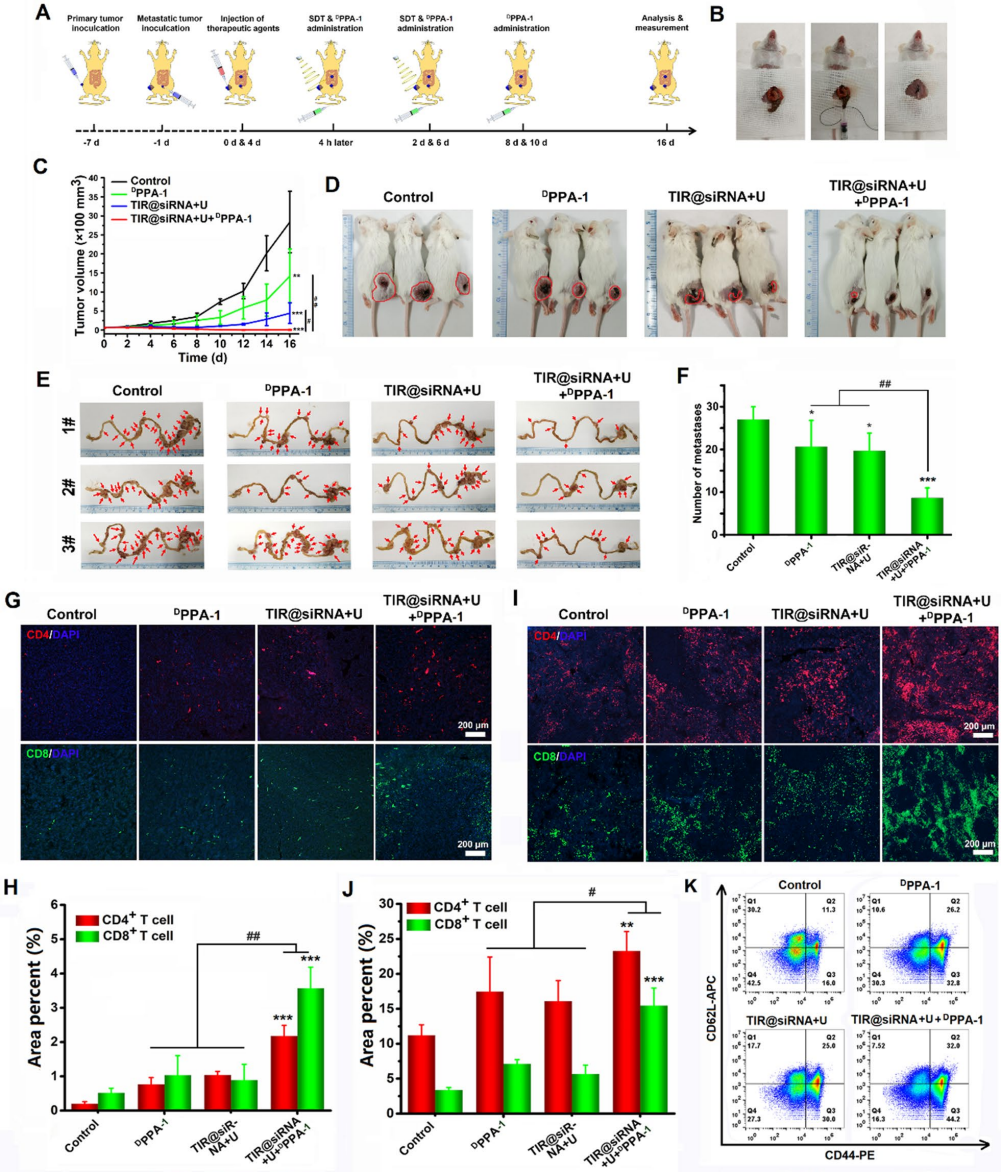
Synergistic anticancer effects of TIR@siRNA mediated SDT combined ^D^PPA-1 peptide mediated anti-PD-L1 therapy in CT26-bearing mice. **A** Therapeutic schedule for subcutaneous and intestinal *in situ* CT26-tumor bearing mice. **B** The photos of implantation of metastatic CT26 colorectal tumor. **C** Tumor growth curves of subcutaneous CT26 tumors during various treatments of normal saline, ^D^PPA-1 peptide, TIR@siRNA with US laser irradiation and TIR@siRNA with US laser irradiation plus ^D^PPA-1 peptide. **D** Photos of the mice at the end of various treatments (the red circles indicate the subcutaneous CT26 tumors). **E** Photos of the intestines dissected from the mice at the end of various treatments (the red arrows indicate the metastatic nodules of CT26 tumors). **F** The counting analysis of metastatic nodules in intestines. Fluorescence images **G** and corresponding semi quantitative analysis **H** of subcutaneous tumor sections with immune-fluorescence staining of CD4 and CD8 at 4 d post treatments. Fluorescence images **I** and corresponding semi quantitative analysis (**J**) of spleen sections with immunofluorescence staining of CD4 and CD8 at the end of treatments. **K** Flow cytometry analysis of CD44 and CD62L expression on splenic T lymphocytes at the end of the treatments. In the experiment, the dosages of IR780, Nrf2-siRNA and ^D^PPA-1 were 2.0 mg/kg, 0.5 mg/kg and 30 mg/kg, respectively. All data are presented as the mean± SD (n = 3). *P < 0.05, **P < 0.01, ***P < 0.005 as compared to the control, ^#^*P* <0.05, ^##^*P* <0.01 for the comparison between two treatment groups

Next, an orthotopic subcutaneous CT26 metastatic colorectal tumor-bearing mouse model was established as mentioned above 1 d before experimental treatment. This tumor model was used to evaluate the antitumor immune response activated by TIR@siRNA-mediated gene-augmented nuclear-targeting SDT combined with ^D^PPA-1 peptide-mediated anti-PD-L1 therapy against colorectal cancer metastasis. The treatments were carried out according to the therapeutic schedule shown in Fig. 9A. Figure 9B visually shows the construction of the metastatic colorectal tumor model. The volumes of the subcutaneous CT26 tumors during the treatment period were measured and are shown in Fig. 9C. The free ^D^PPA-1 peptide and TIR@siRNA combined with US irradiation both exhibited evident inhibition of subcutaneous CT26 tumors, while combination therapy consisting of TIR@siRNA-based SDT and ^D^PPA-1 peptide-mediated anti-PD-L1 therapy displayed the greatest ablation effects on CT26 tumors. The *in situ* tumor tissues (Fig. 9D) and dissected tumor tissues (Additional file 1: Fig. S7) images taken at the end of the various treatments further proved the above results. Moreover, the intestines of the mice were harvested to observe metastatic nodules, and these images are shown in Fig. 9E. Compared to the control, all three treatments exhibited inhibitory effects on metastasis to different extents, while the combinatory therapy showed the best performance. Counting analysis (Fig. 9F) further indicated the strongest inhibitory effect of the combinatory treatment. The tumor suppression rates of treatment with the free ^D^PPA-1 peptide, TIR@siRNA with US irradiation and TIR@siRNA with US irradiation combined with the ^D^PPA-1 peptide were 23.5%, 27.2% and 67.9%, respectively. Body weight changes were monitored during the treatment period, and no reduction in body weight was observed, as shown in Additional file 1: Fig. S8, suggesting the safety of this combination treatment. This synergistic antitumor efficacy was repeated evaluated under same conditions, the similar curative effect was acquired and shown in Additional file 1: Fig. S9. To further elucidate the antitumor efficacy of gene-augmented nuclear-targeting SDT boosted anti-PD-L1 therapy, we assessed the infiltration of helper CD4^+^ T cells and cytotoxic CD8^+^ T cells in the subcutaneous tumor tissues and spleen tissues after antigen presentation by DCs. Figure 9G, H shows the CD4^+^ T cells and CD8^+^ T cells in subcutaneous tumor tissues on day 4 after the various treatments. The infiltration rates of CD4^+^ T cells and CD8^+^ T cells were 0.20%, 0.77%, 1.04%, and 2.18% and 0.52%, 1.03%, 0.89%, and 3.57% in the control, free ^D^PPA-1 peptide, TIR@siRNA with US irradiation and combination treatment groups, respectively. Furthermore, the antitumor immune cytokines (IFN-γ and TNF-α) expressed CD4^+^ and CD8^+^ T cells in tumor tissues were also evaluated and shown in Additional file 1: Fig. S10. Clearly, this combination treatment evidently enhanced the infiltration of IFN-γ^+^, TNF-α^+^ CD4^+^ T cells and IFN-γ^+^, TNF-α^+^ CD8^+^ T cells, which indicated that the immune response was stimulated by SDT and anti-PD-L1 therapy to eradicate the solid tumor. The number of CD4^+^ T cells and CD8^+^ T cells in the spleen tissues were determined and these results are shown in Fig. 9I, J. The combination treatment exhibited maximum immune CD4^+^ T cell and CD8^+^ T cell infiltration compared to other treatments, which showed that the systemic immune system was activated to suppress CT26 tumor metastasis. To investigate the specific antitumor immune memory effect, the memory T cells like CD44^+^CD62L^−^ in spleen were investigated and shown in Fig. 9K. The CD44^+^CD62L^−^ T cells were up to 44.2% of T cells in spleen at the end of combination treatment, which were higher than that in the individually TIR@siRNA+U (30.0%) and ^D−^PPA peptide (32.8%) treatment groups. Above results validated that TIR@siRNA-mediated gene-augmented nuclear-targeting SDT could improve the immunosuppressive microenvironment to boost ^D^PPA-1 peptide-based anti-PD-L1 therapy to eliminate colorectal cancer and inhibit its metastasis.

## Conclusions

In this study, we successfully prepared TIR@siRNA nanoparticles with the nuclear-targeting sonosensitizer TIR loaded with Nrf2-siRNA. TIR@siRNA greatly improved the internalization and nuclear localization of IR780 by conjugation with the TAT peptide and codelivery of Nrf2-siRNA. TIR@siRNA showed excellent gene-augmented nuclear-targeting SDT against colorectal cancer in vitro and in vivo by blocking activation of the Nrf2-based antioxidant pathway. Gene-augmented nuclear-targeting SDT significantly induced ICD in colorectal cancer, thus boosting ^D^PPA-1 peptide-based anti-PD-L1 therapy to ablate the primary tumor and suppress metastatic tumors. Furthermore, TIR@siRNA showed extended tumor retention and fluorescence imaging and PA imaging properties. These results suggested that TIR@siRNA could be a SDT agent for colorectal cancer treatment and is promising for the facilitation of ICB therapy in colorectal cancer.

## Supplementary Information

**Additional file 1: Fig. S1.** Thin-layer chromatography image of IR780, TAT-IR780 and TAT mixed IR780 (TAT/IR780). The mass spectra of TAT-IR780. **Fig. S2.** Optical stabilities of IR780, TIR and TIR@siRNA nanoparticles. **Fig. S3.** The digital images of CT26 spheroids. **Fig. S4.** Evaluation of lysosomal permeabilization by acridine orange. **Fig. S5.** PA imaging efficiency of TIR@siRNA. **Fig. S6.** Fluorescence microscopic images of main organs from the mice after various treatments. **Fig. S7.** The images of isolated tumors at the end of various treatments. **Fig. S8.** Body weight changes during various treatments. **Fig. S9.** The repeated investigation of synergistic anticancer effects of TIR@siRNA mediated SDT combined DPPA-1 peptide mediated anti-PD-L1 therapy in CT26-bearing mice. **Fig. S10.** Immunofluorescence histochemical analysis of IFN-γ+CD4+ T cells, TNF-α+ CD4+ T cells and IFN-γ+CD8+ T cells, TNF-α+CD8+ T cells in tumor tissues at 4 d after various treatments.

## Data Availability

All data analyzed during this study are included in this published article and its supplementary information files.
