# Supplementary material for: Gene augmented nuclear-targeting sonodynamic therapy via Nrf2 pathway-based redox balance adjustment boosts peptide-based anti-PD-L1 therapy on colorectal cancer
Source: J Nanobiotechnology. 2021 Oct 29;19:347. doi: 10.1186/s12951-021-01094-x (PMC8555306; doi:10.1186/s12951-021-01094-x)
Supplement: Supplementary file 1 — Additional file 1: Fig. S1. Thin-layer chromatography image of IR780, TAT-IR780 and TAT mixed IR780 (TAT/IR780). The mass spectra of TAT-IR780. Fig. S2. Optical stabilities of IR780, TIR and TIR@siRNA nanoparticles. Fig. S3. The digital images of CT26 spheroids. Fig. S4. Evaluation of lysosomal permeabilization by acridine orange. Fig. S5. PA imaging efficiency of TIR@siRNA. Fig. S6. Fluorescence microscopic images of main organs from the mice after various treatments. Fig. S7. The images of isolated tumors at the end of various treatments. Fig. S8. Body weight changes during various treatments. Fig. S9. The repeated investigation of synergistic anticancer effects of TIR@siRNA mediated SDT combined DPPA-1 peptide mediated anti-PD-L1 therapy in CT26-bearing mice. Fig. S10. Immunofluorescence histochemical analysis of IFN-γ+CD4+ T cells, TNF-α+ CD4+ T cells and IFN-γ+CD8+ T cells, TNF-α+CD8+ T cells in tumor tissues at 4 d after various treatments. [file 12951_2021_1094_MOESM1_ESM.docx]

**Additional Material**

**Gene** **augmented nuclear-targeting sonodynamic therapy via Nrf2 pathway-based redox balance adjustment boosts peptide-based anti-PD-L1 therapy on colorectal cancer**

Guoyun Wan^1,†,^*, Xuheng Chen^1,†^, Haijiao Wang^1,†^, Shenglei Hou^3^, Qian Wang^1^, Yuanyuan Cheng^2^, Qian Chen^2^, Yingge Lv^1^, Hongli Chen^1,^*, Qiqing Zhang^1,3,^*

^1^ *The Key Laboratory of Biomedical Material, School of Life Science and Technology,* *Xinxiang Medical University, Xinxiang 453003, China*

^2^ *School of Pharmacy, Tianjin Key Laboratory on Technologies Enabling Development of Clinical Therapeutics and Diagnostics (Theranostics), Tianjin Medical University, Tianjin 300070, China*

^3^*Institute of Biomedical Engineering, The Second Clinical Medical College, Jinan University (Shenzhen People's Hospital), Shenzhen 518020, China; Post-doctoral Scientific Research Station of Basic Medicine, Jinan Unviersity, Guangzhou 510632, China*

^†^ These authors contributed equally to this work.

* Corresponding authors: E-mail: [wanguoyun@xxmu.edu.cn](mailto:wanguoyun@xxmu.edu.cn) (G. Wan); [chenhl@xxmu.edu.cn](mailto:chenhl@xxmu.edu.cn) (H. Chen); [zhangqiq@126.com](mailto:zhangqiq@126.com) (Q. Zhang).


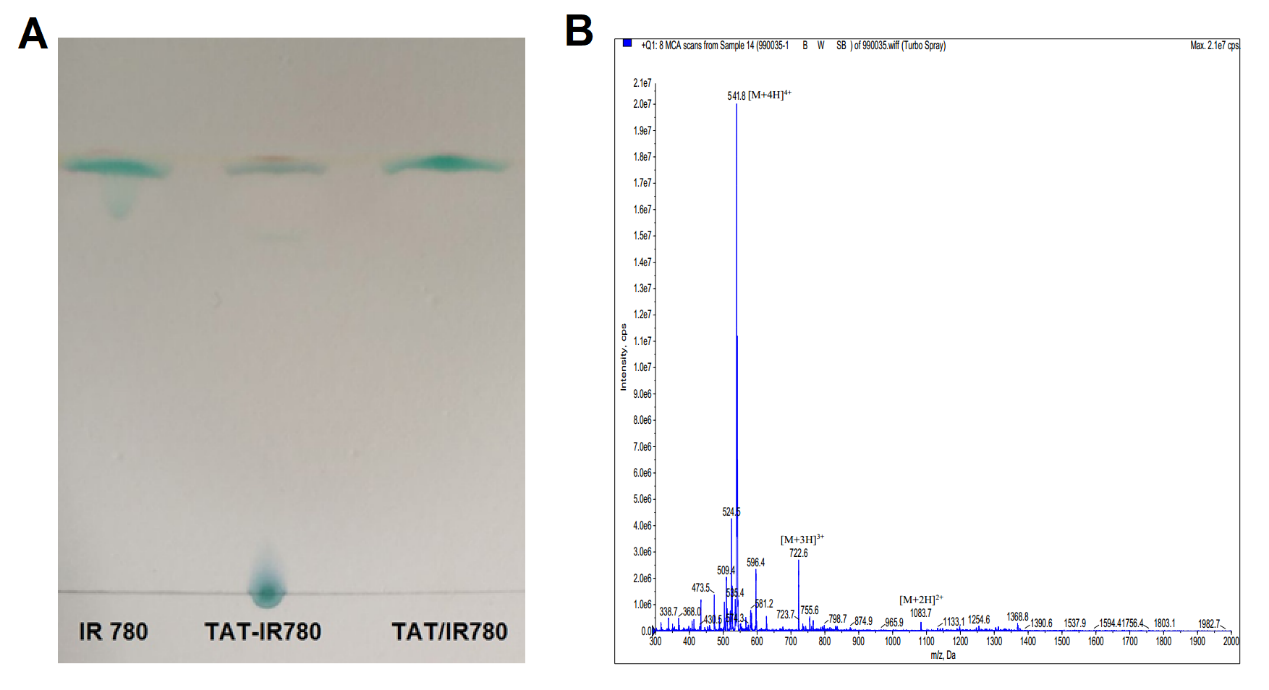


**Fig. S1.** (A) Thin-layer chromatography image of IR780, TAT-IR780 and TAT mixed IR780 (TAT/IR780). (B) The mass spectra of TAT-IR780.


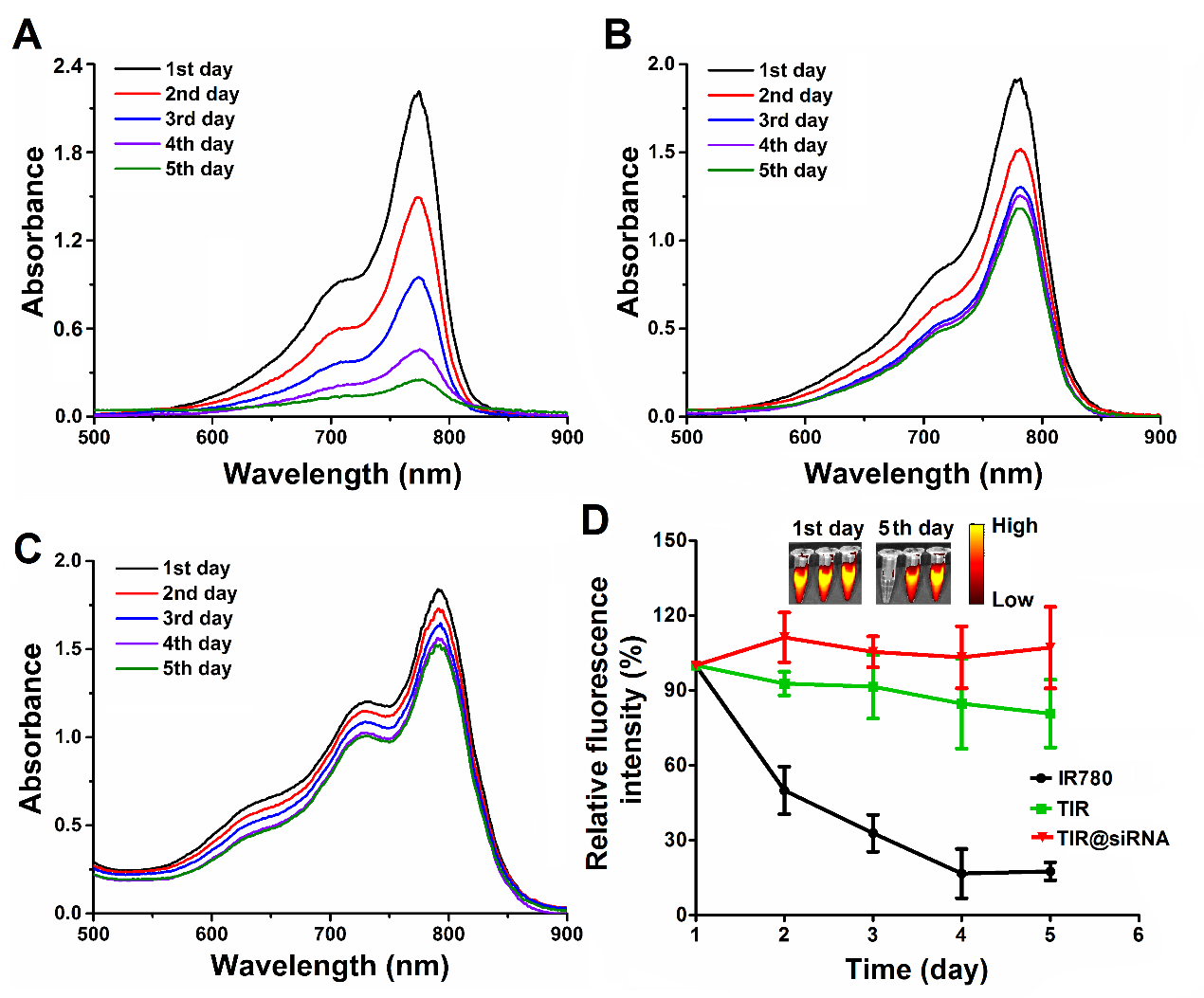


**Fig. S2.** Optical stabilities of IR780, TIR and TIR@siRNA nanoparticles. UV-vis absorption spectra of IR780 (A), TIR (B) and TIR@siRNA (C) during 5-day storage in DEPC water at IR780 concentration of 10 μg/mL. (D) Fluorescence intensity changes of IR780, TIR and TIR@siRNA during 5-day storage in DEPC water at IR780 concentration of 2 μg/mL (the insert images are the fluorescence images of IR780, TIR and TIR@siRNA (from left to right) on 1^st^ and 5^th^ day).


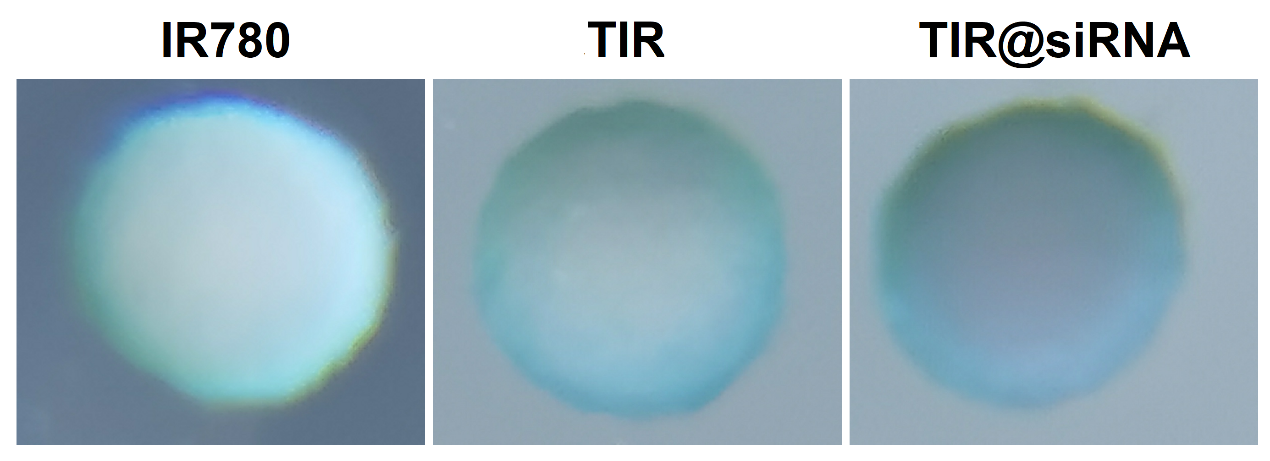


**Fig. S3.** The digital images of CT26 spheroids after 12-h incubation of IR780, TIR and TIR@siRNA.


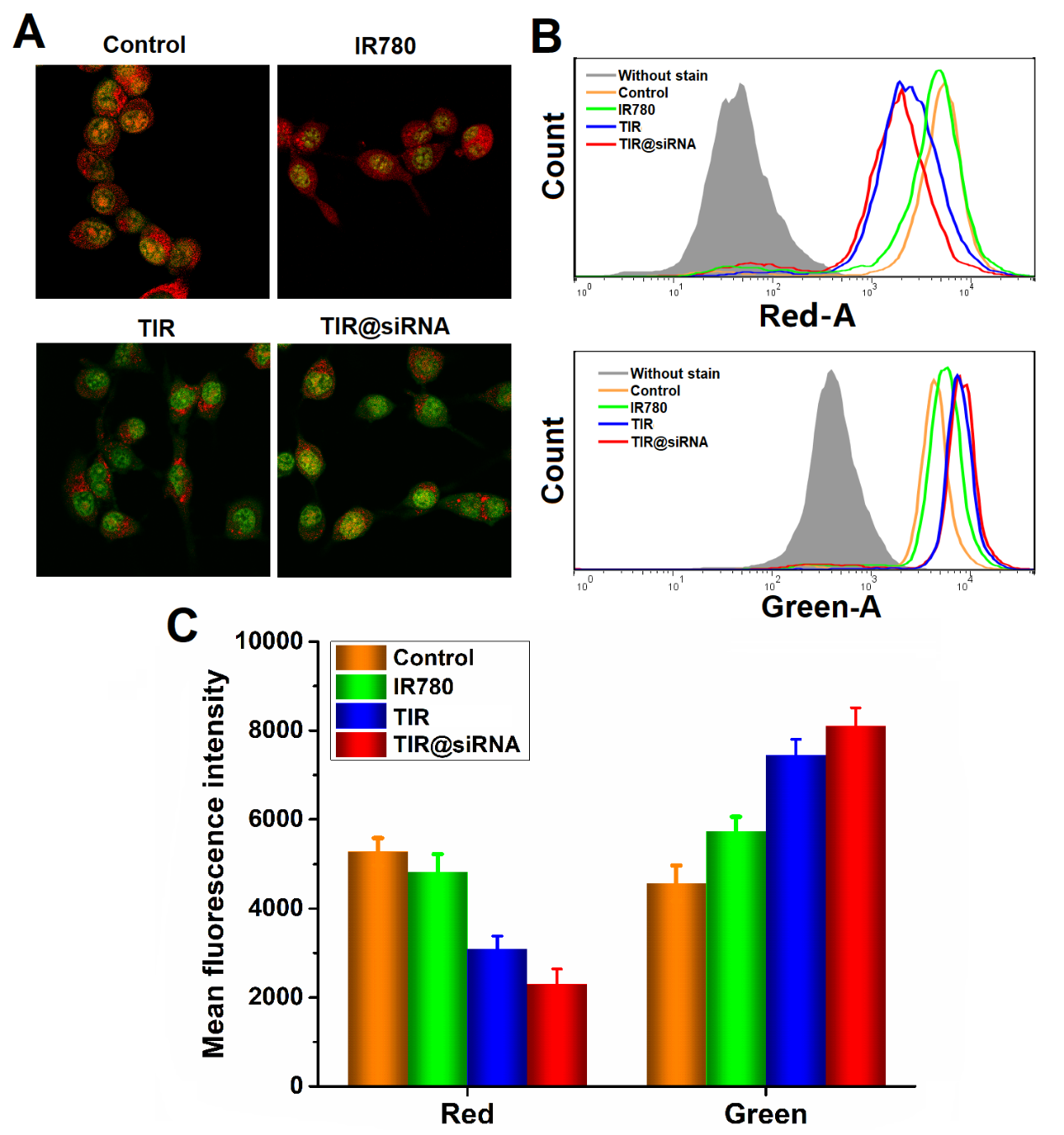


**Fig. S4.** Evaluation of lysosomal permeabilization by acridine orange. (A) Confocal images, (B) flow cytometry curves and (C) quantitative analysis of red fluorescence and green fluorescence in acridine orange stained CT26 cells after 4 h incubation with IR780, TIR and TIR@siRNA.


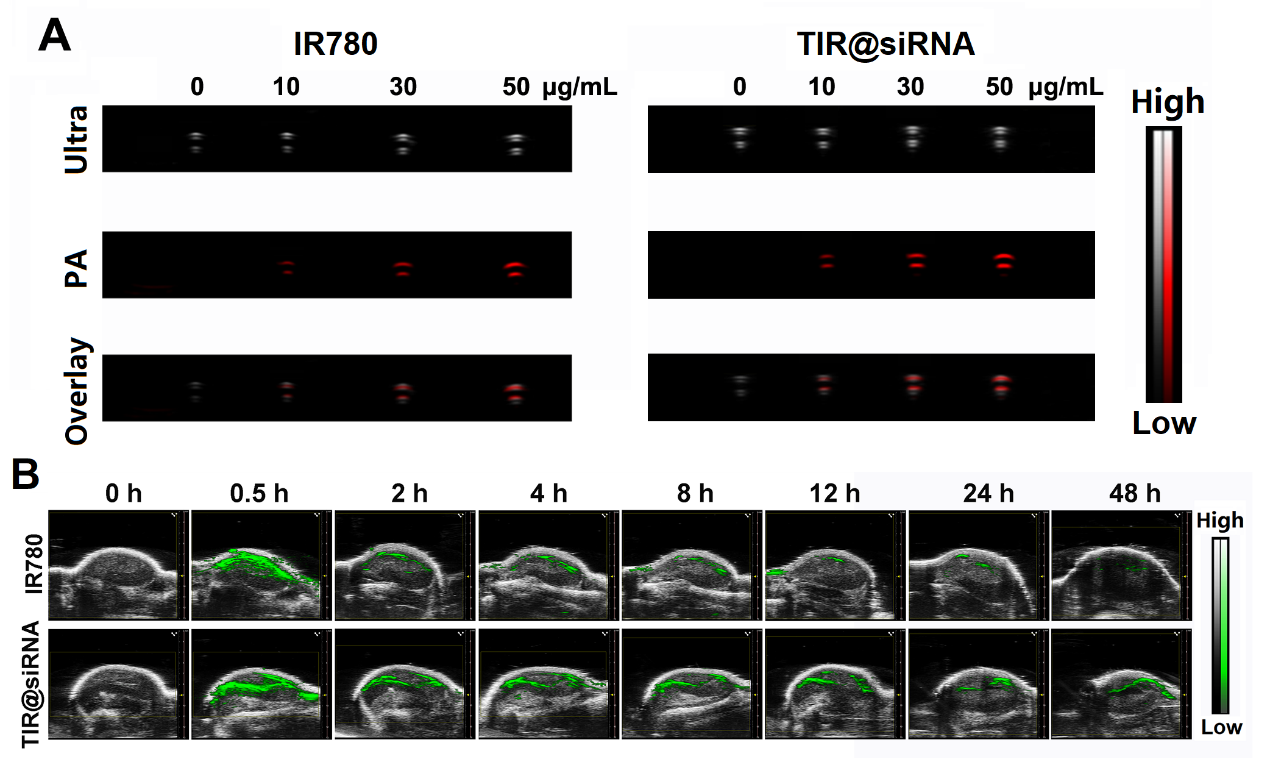


**Fig. S5.** PA imaging efficiency of TIR@siRNA. (A) *In vitro* PA images of free IR780 and TIR@siRNA at various concentrations. (B) *In vivo* PA images of the tumor tissues at different time points post intratumoral injection of free IR780 and TIR@siRNA nanoparticles. “0 h” represents pre-injection.


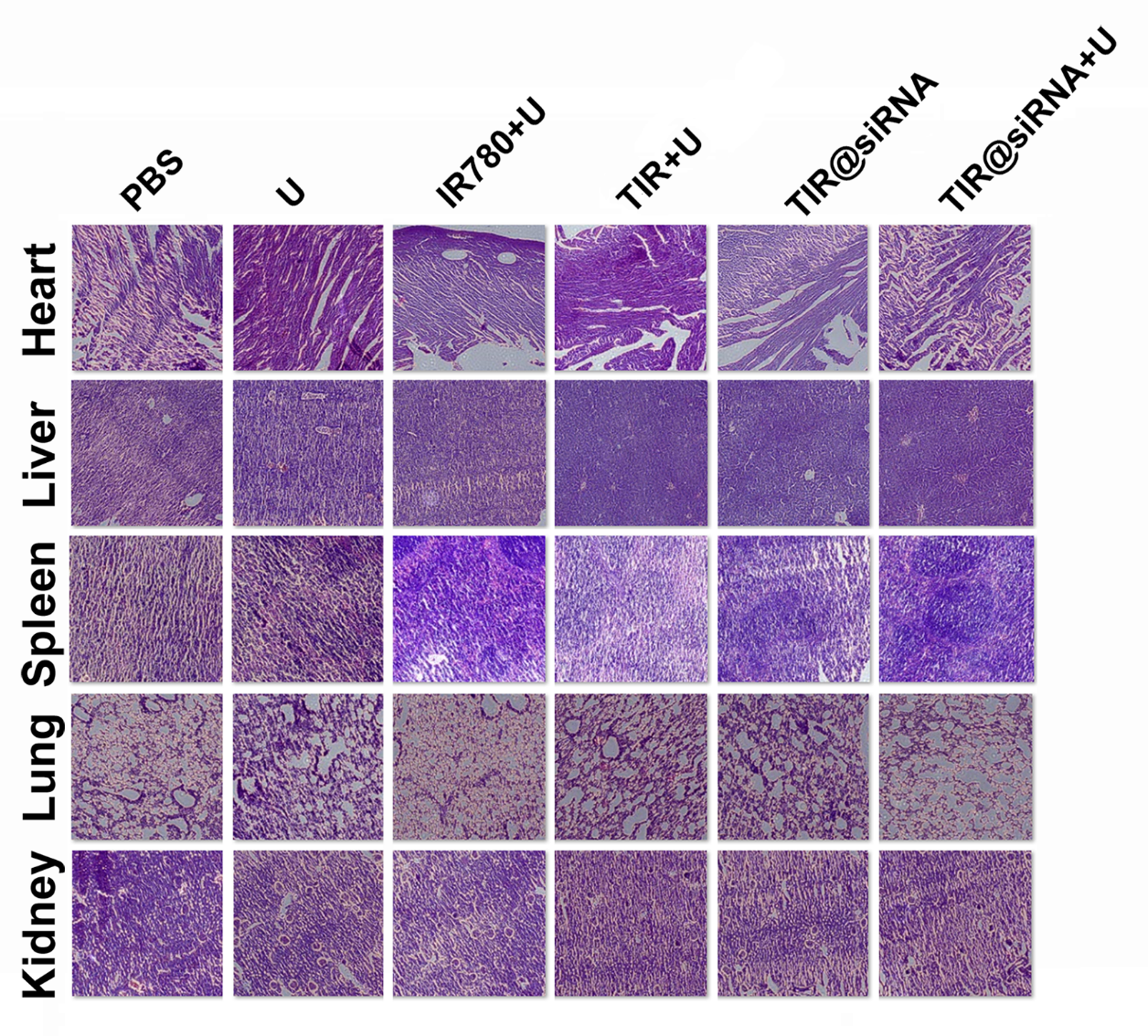


**Fig. S6.** Fluorescence microscopic images of main organs from the mice after various treatments.


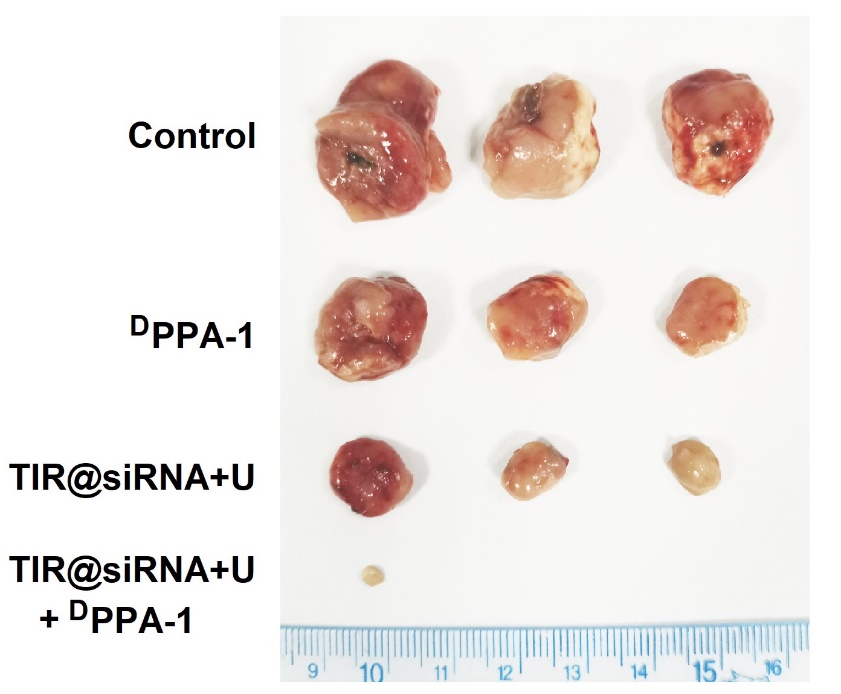


**Fig. S7.** The images of isolated tumors at the end of various treatments.


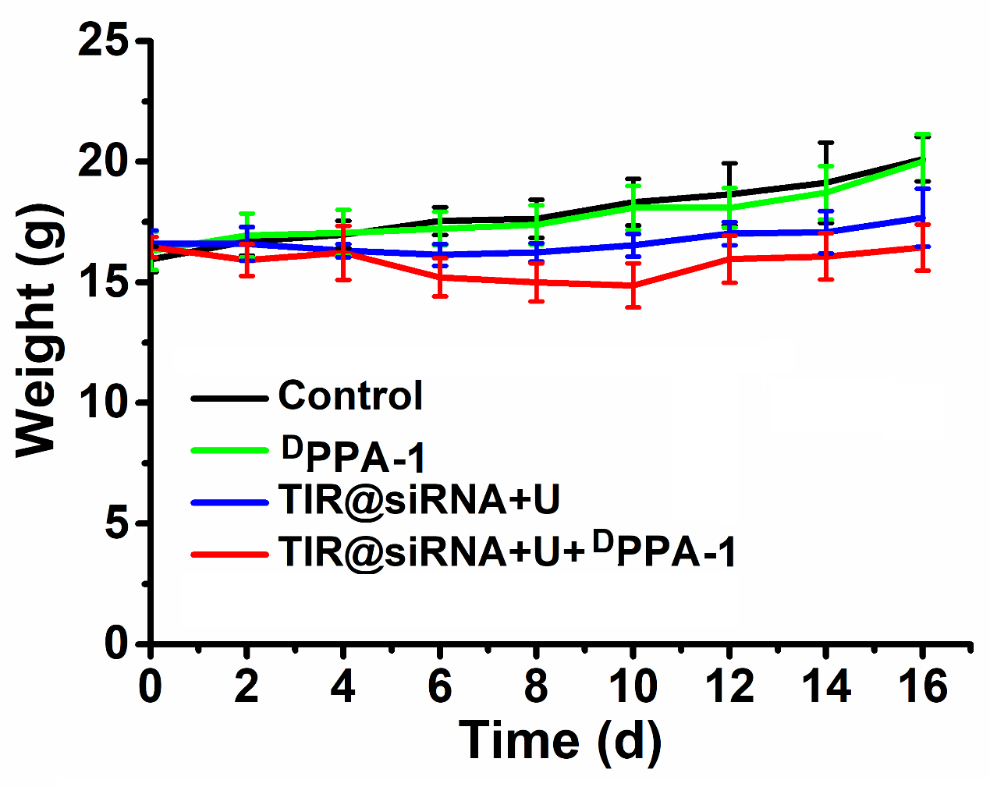


**Fig. S8.** Body weight changes during various treatments.


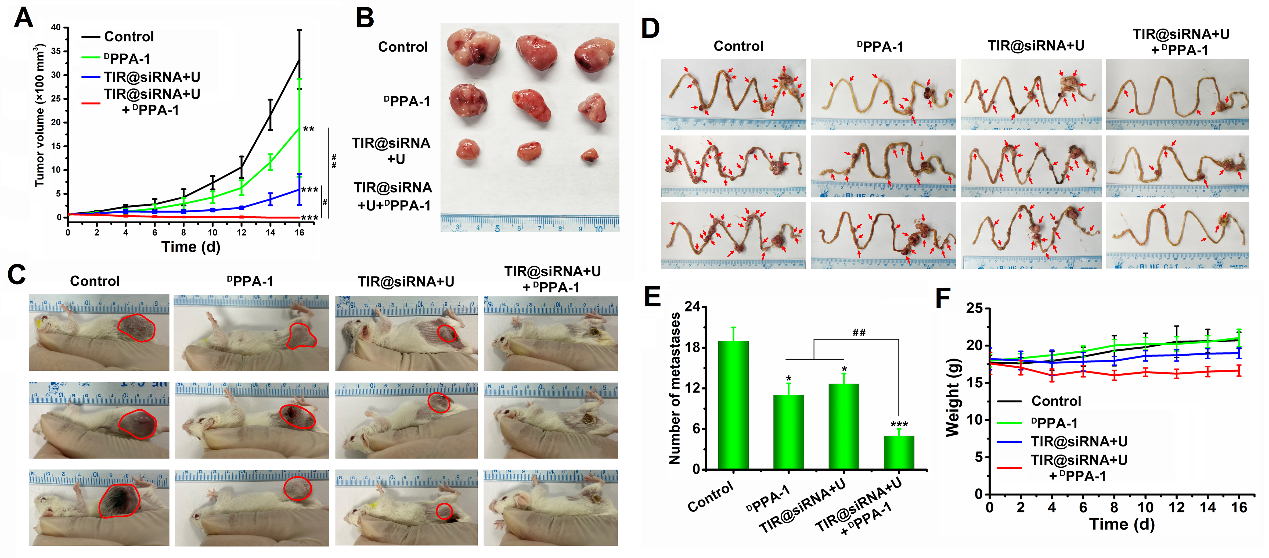


**Fig. S9.** The repeated investigation of synergistic anticancer effects of TIR@siRNA mediated SDT combined ^D^PPA-1 peptide mediated anti-PD-L1 therapy in CT26-bearing mice. (A) Tumor growth curves of subcutaneous CT26 tumors during various treatments of normal saline, ^D^PPA-1 peptide, TIR@siRNA with US laser irradiation and TIR@siRNA with US laser irradiation plus ^D^PPA-1 peptide. (B) The images of isolated tumors at the end of various treatments. (C) Photos of the mice at the end of various treatments (the red circles indicate the subcutaneous CT26 tumors). (D) Photos of the intestines dissected from the mice at the end of various treatments (the red arrows indicate the metastatic nodules of CT26 tumors). (E) The counting analysis of metastatic nodules in intestines. (F) The body weight change curves of the mice during the treatments. In the experiment, the dosages of IR780, Nrf2-siRNA and ^D^PPA-1 were 2.0 mg/kg, 0.5 mg/kg and 30 mg/kg, respectively. All data are presented as the mean± SD (n = 3). **P* < 0.05, ***P* < 0.01, ****P* < 0.005 as compared to the control, *^#^P* <0.05, *^##^P* <0.01 for the comparison between two treatment groups.


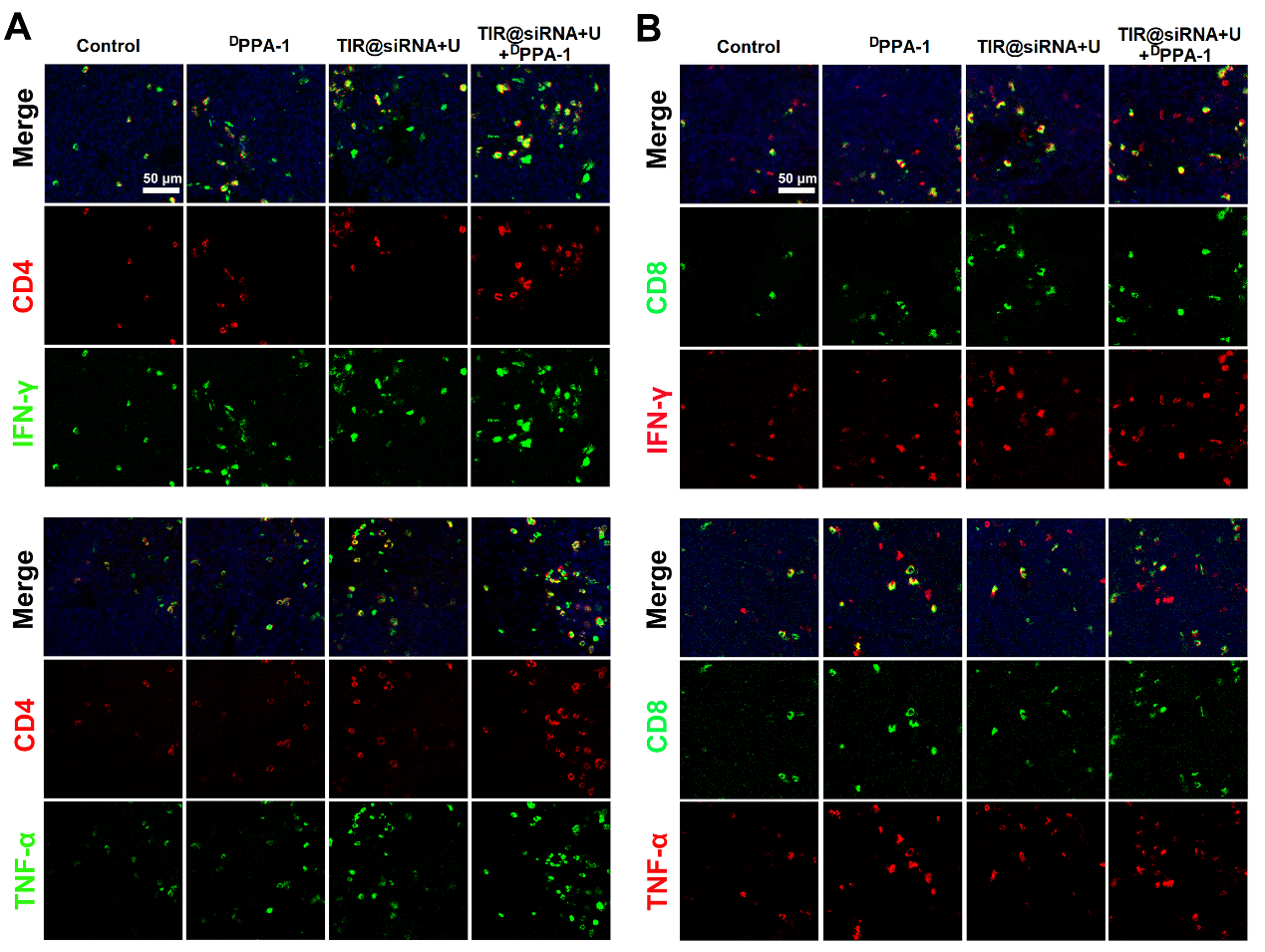


**Fig. S10.** Immunofluorescence histochemical analysis of IFN-γ^+^CD4^+^ T cells, TNF-α^+^ CD4^+^ T cells (A) and IFN-γ^+^CD8^+^ T cells, TNF-α^+^CD8^+^ T cells (B) in tumor tissues at 4 d after various treatments.
